# Supplementary figures and images for: Autotoxin-mediated latecomer killing in yeast communities
Source: PLoS Biol. 2022 Nov 7;20(11):e3001844. doi: 10.1371/journal.pbio.3001844 (PMC9639812; doi:10.1371/journal.pbio.3001844)

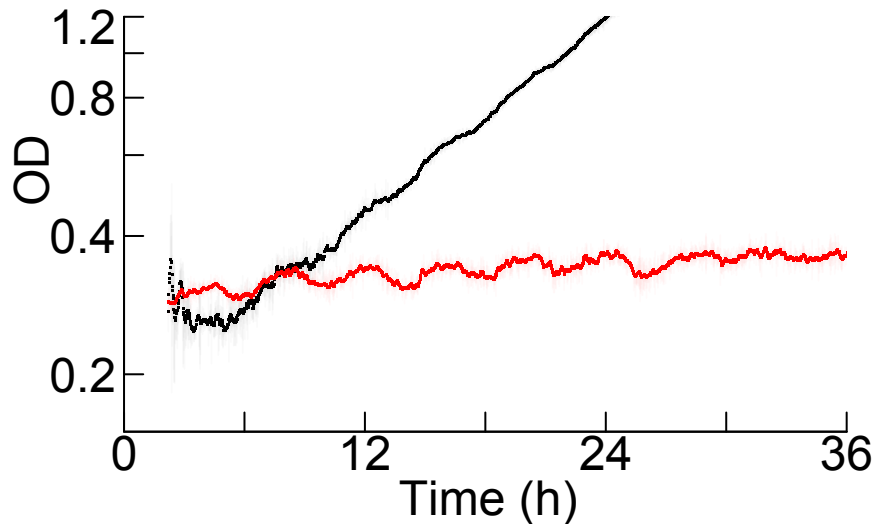

— MM with 3% Glycerol  
— MM without Glycerol

Supplement: S1 Fig — Growth curves of WT cells in the MM without glucose and with 3% glycerol (black) and that without both glucose and glycerol (red). Cells were precultured in the MM with 3% glucose and 3% glycerol. Each line represents an average of n = 2 samples. The data underlying this figure can be found in S2 Data. (PDF) [file pbio.3001844.s001.pdf]

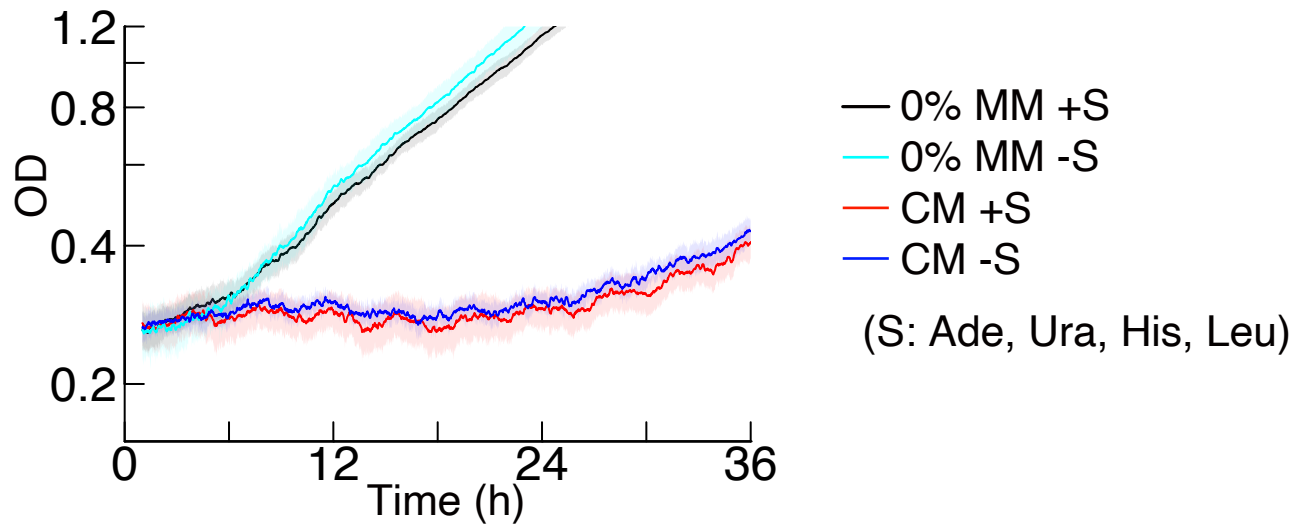

Supplement: S2 Fig — Growth curves of WT cells in the MM with and without leucine, adenine, uracil, and histidine, and those media conditioned by the WT cell. Each line represents an average of n = 4–8 samples. The data underlying this figure can be found in S2 Data. (PDF) [file pbio.3001844.s002.pdf]

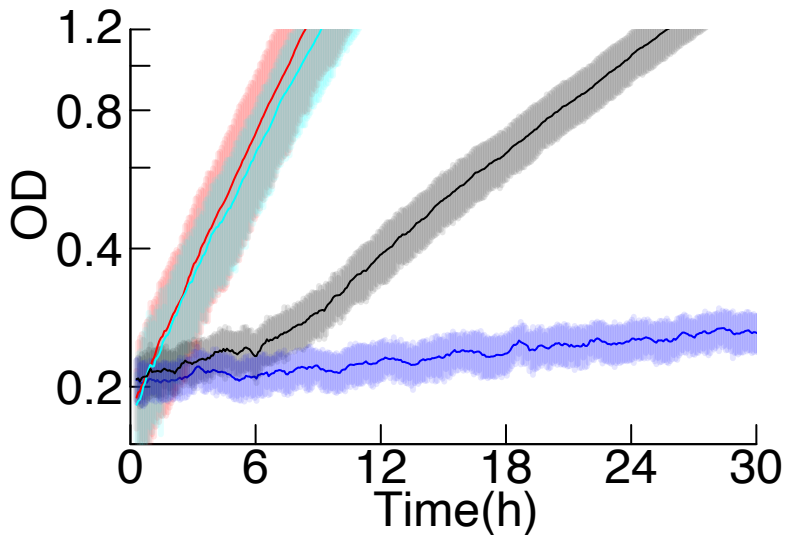

— WT in 3% MM    — *fbp1*Δ in 3% MM  
— WT in 0% MM    — *fbp1*Δ in 0% MM

Supplement: S3 Fig — fbp1Δ and WT cells were precultured in 3% MM and then transferred to 0% or 3% MM. Growth curves of fbp1Δ cells in 0% and 3% MM are shown in blue and light blue, respectively, and those of WT cells are shown in black and red, respectively. Each line represents an average of 3–7 samples. The data underlying this figure can be found in S2 Data. (PDF) [file pbio.3001844.s003.pdf]

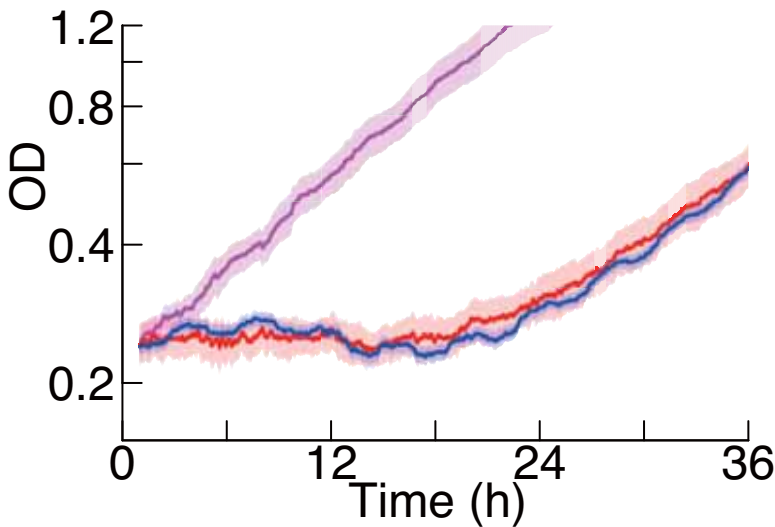

- CM survivor
- Original cells
- 0% MM-adapted cells

Supplement: S5 Fig — The blue line shows the average growth curves of cells in 24 independent colonies isolated from surviving cells in WT CM. Red and magenta lines are the average growth curves of original WT cells precultured in 3% and 0% MM, respectively. The data underlying this figure can be found in S2 Data. (PDF) [file pbio.3001844.s005.pdf]

**A**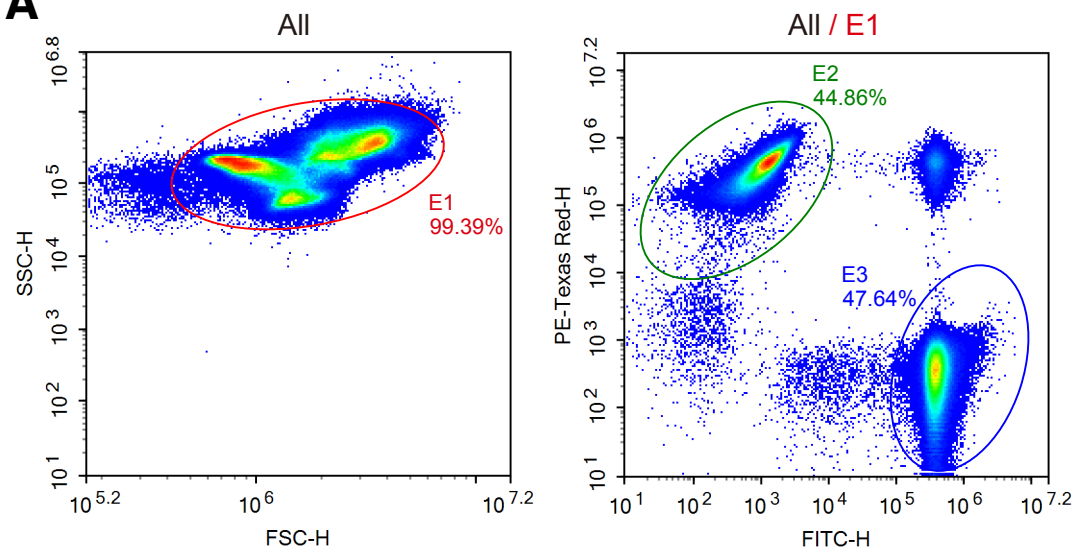**B**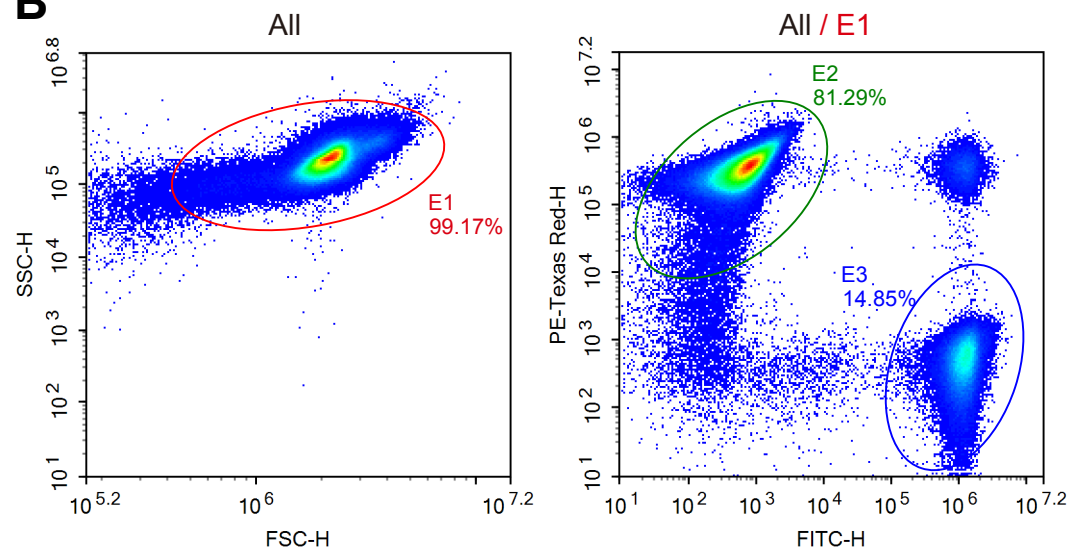**C**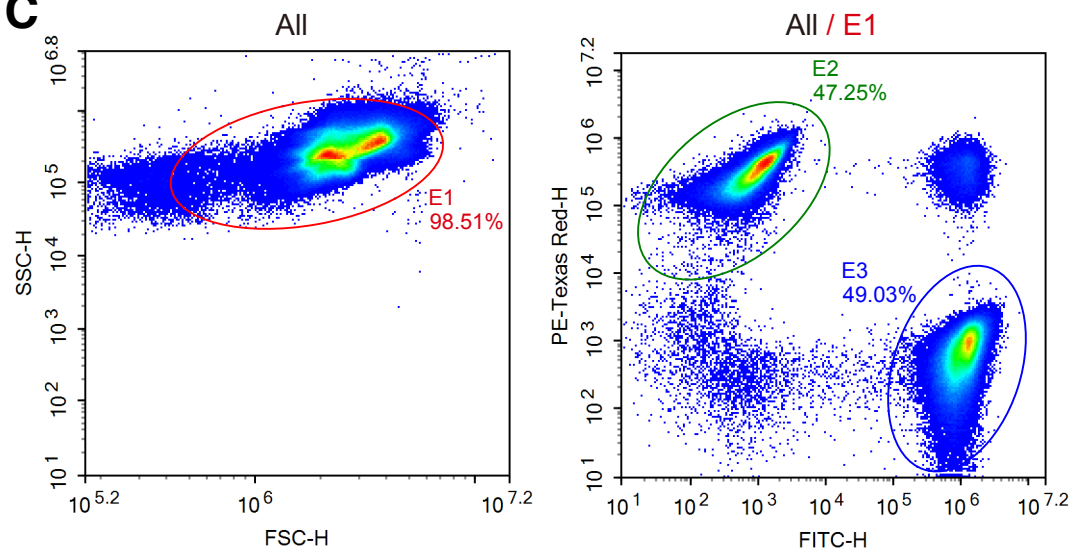**D**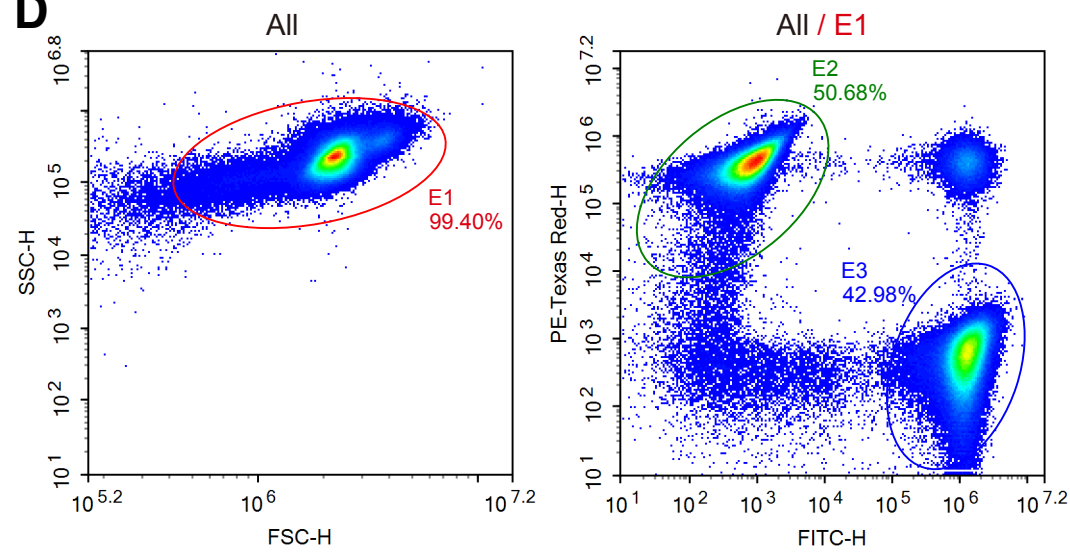

Supplement: S7 Fig — (A-D) Fission yeast cells were first gated on the red E1 gate (left panel). Then, cells in the E2 area were defined as mCherry-positive cells and cells in the E3 area were defined as mNeon-Green-positive cells (right panel). The total count of each sample is 500,000 counts. (A) An example image of competition assay between mCherry-tagged cells precultured in 0% MM and mNeonGreen-tagged cells precultured in 3% MM at 0 h. (B) An example image of competition assay between mCherry-tagged cells precultured in 0% MM and mNeonGreen-tagged cells precultured in 3% MM at 72 h. (C) An example image of competition assay between mCherry-tagged cells precultured in 0% MM and mNeonGreen-tagged cells precultured in 0% MM at 0 h. (D) An example image of competition assay between mCherry-tagged cells precultured in 0% MM and mNeonGreen-tagged cells precultured in 0% MM at 72 h. (PDF) [file pbio.3001844.s007.pdf]

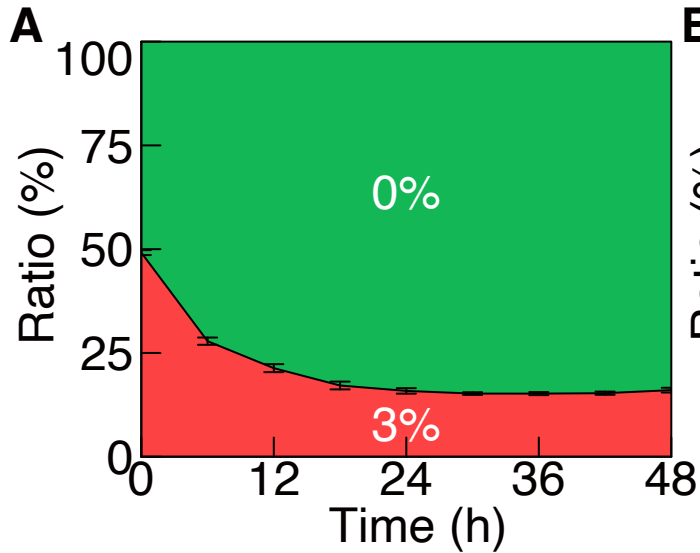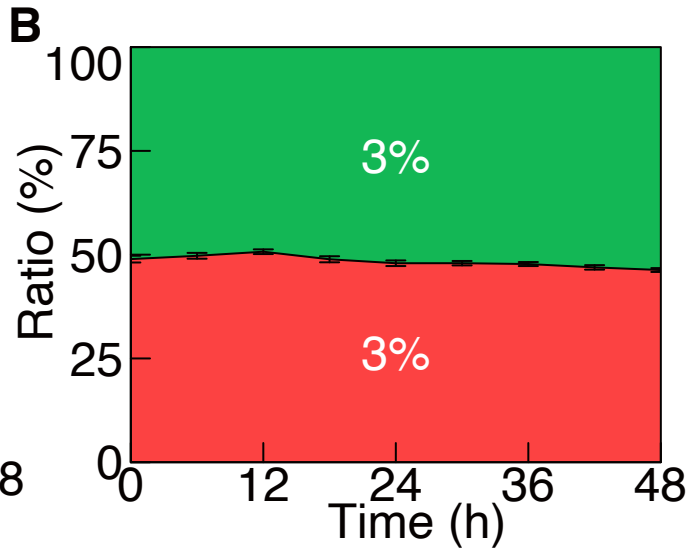

Supplement: S8 Fig — mNeonGreen- and mCherry-labelled WT cells were mixed in equal fractions in the WT CM at 0 h, and then they showed population dynamics. Green and red areas indicate the fraction of mNeonGreen- and mCherry-labelled cells, respectively, and overwriting outline characters indicate preculture conditions, i.e., 3% and 0% indicate cells precultured in 3% and 0% MM, respectively. Black vertical bars between 2 areas indicate SEM (number of each sample is 12). (A) Competition assay between mNeonGreen-labelled cells precultured in 0% MM and mCherry-labelled cells precultured in 3% MM. (B) Competition assay between mNeonGreen- and mCherry-labelled cells precultured in 3% MM. The data underlying this figure can be found in S1 Data. (PDF) [file pbio.3001844.s008.pdf]

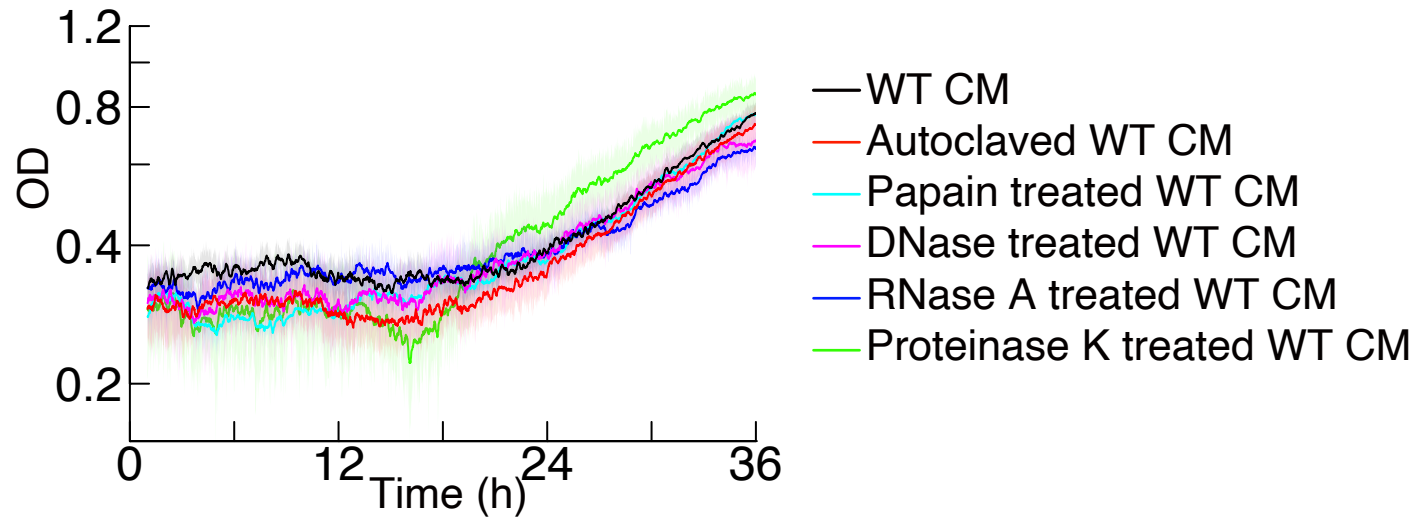

Supplement: S9 Fig — WT cells were precultured in 3% MM, then growth was measured in the various CM treated as follows: Autoclaved: WT CM was autoclaved at 121°C for 20 min. Papain treated: WT CM was treated with 2.5 mg/ml papain (Cat#166–00171, Wako) at 37°C for 24 h. RNase A treatment: WT CM was treated with 50 μg/ml RNaseA (Cat#318–06391, Nippon gene) at 37°C for 24 h. DNase treatment: WT CM was treated with 100 U/ml DNase I (Cat#18047019, Invitrogen) at 37°C for 24 h. Proteinase K treatment: WT CM was treated with 8 μg/ml Proteinase K (Cat# 25530049, Invitrogen) at 37°C for 24 h. Each line represents an average of n = 2–4 samples. The data underlying this figure can be found in S2 Data. (PDF) [file pbio.3001844.s009.pdf]

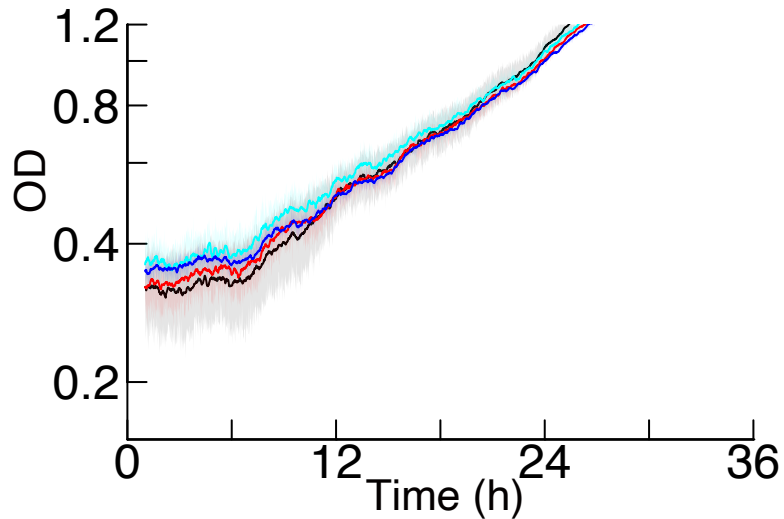

- 0% MM -S
- 0% MM -S + 30 mM Leu
- 0% MM -S + 60 mM Leu
- 0% MM -S + 30 mM BCAAs

(S: Ade, Ura, His, Leu  
BCAAs: Leu, Val, Ile)

Supplement: S11 Fig — Growth curves of WT cells in 0% MM without auxotrophic marker supplements with 30 mM, 60 mM leucine or mixture of branched chain amino acids (BCAA: 30 mM leucine + 30 mM valine + 30 mM isoleucine). WT cells were precultured in 3% MM without auxotrophic marker supplements. Each line represents an average of n = 2 samples. The data underlying this figure can be found in S2 Data. (PDF) [file pbio.3001844.s011.pdf]

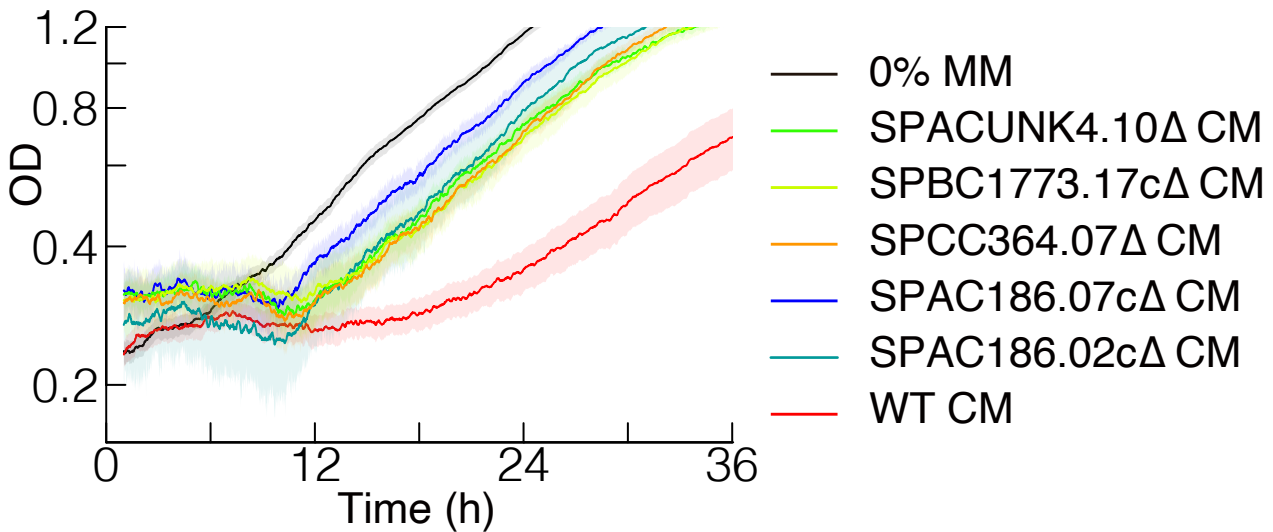

Supplement: S12 Fig — Different colored lines indicate growth curves of WT in media conditioned by different deletion mutants. Each deleted gene is predicted as putative hydroxyacid dehydrogenase or hydroxyacid dehydrogenase homolog in the UniProt database. WT cells were precultured in 3% MM. Each line represents an average of n = 4–7 samples. The data underlying this figure can be found in S2 Data. (PDF) [file pbio.3001844.s012.pdf]

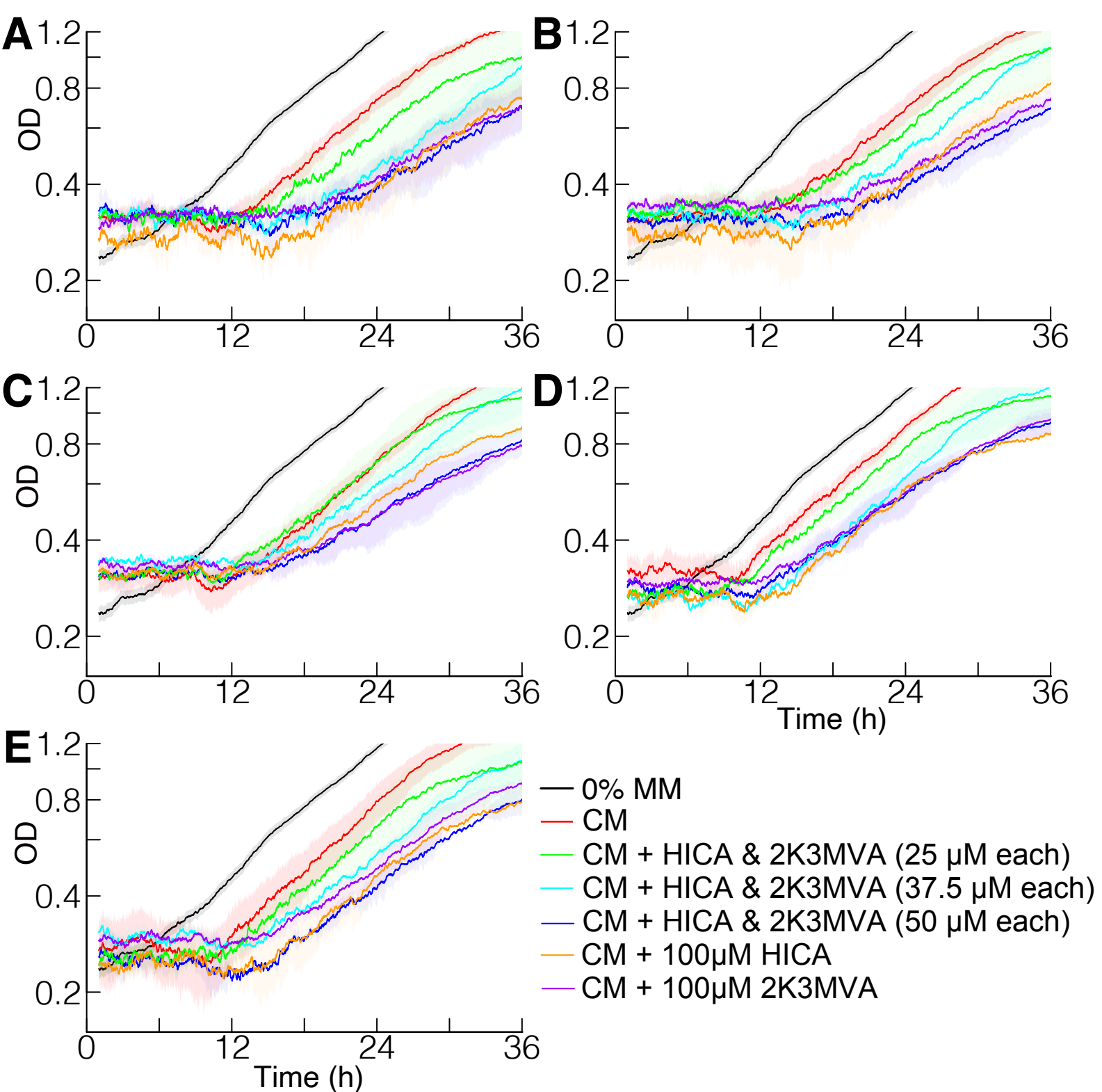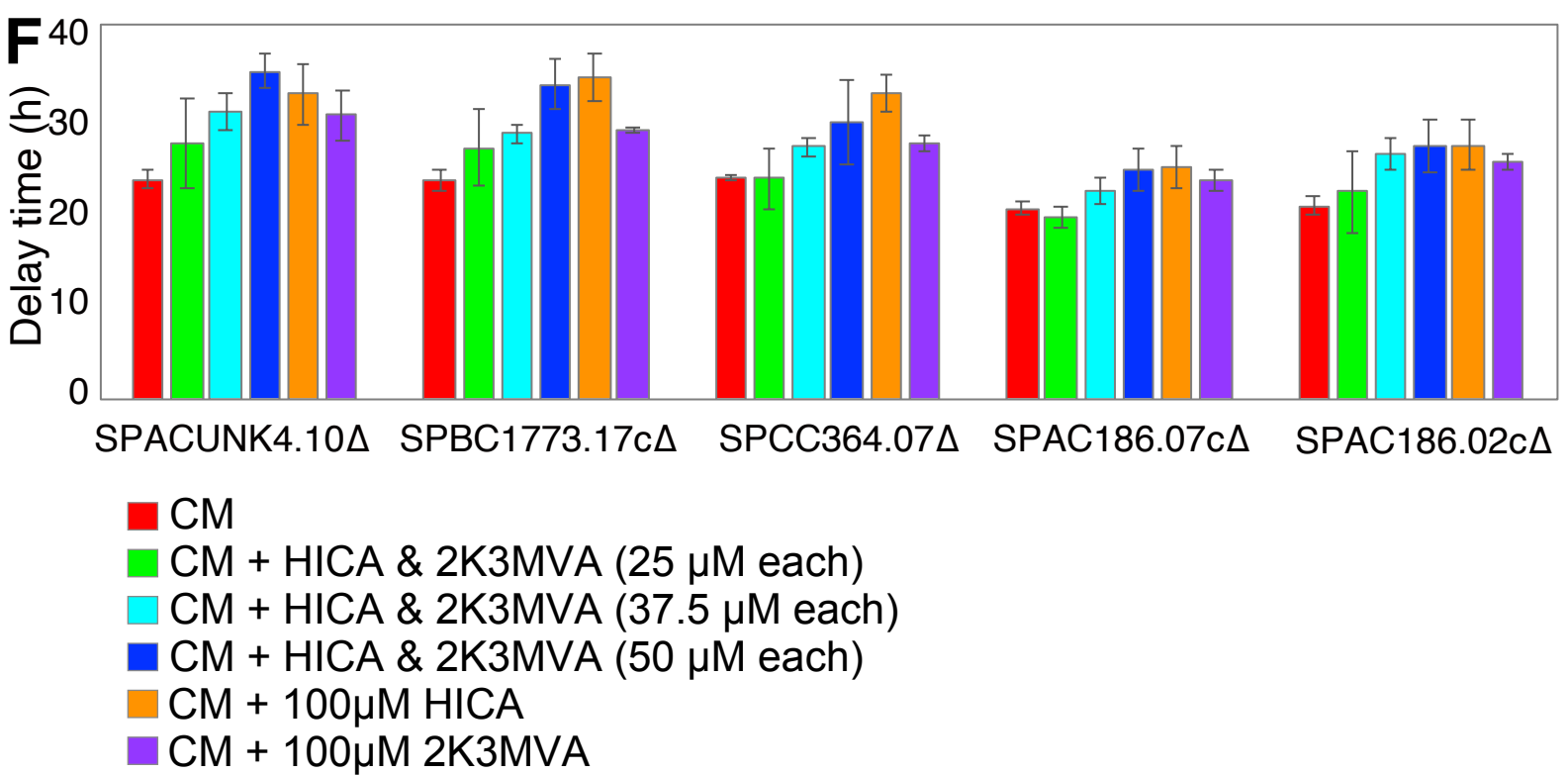

Supplement: S13 Fig — Cells precultured in 3% MM were inoculated to media conditioned by (A) SPACUNK4.10Δ, (B) SPBC1773.17cΔ, (C) SPCC364. 07Δ, (D) SPAC186.07cΔ, and (E) SPAC186.02Δ mutants. The conditioned media from those cultures were added with HICA or 2K3MVA or both of them. (F) Length of the delay phase τ for each growth curve. A line for each conditioned media represents an average of n = 4–7 samples, and those for conditioned media with the autotoxins represents an average of n = 2. The data underlying this figure can be found in S2 Data. (PDF) [file pbio.3001844.s013.pdf]

**A**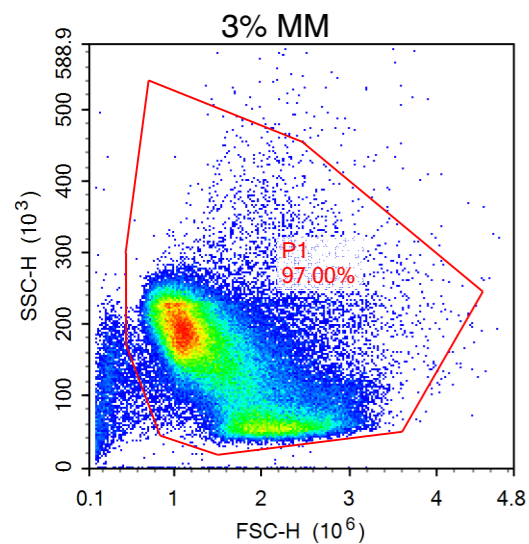**B**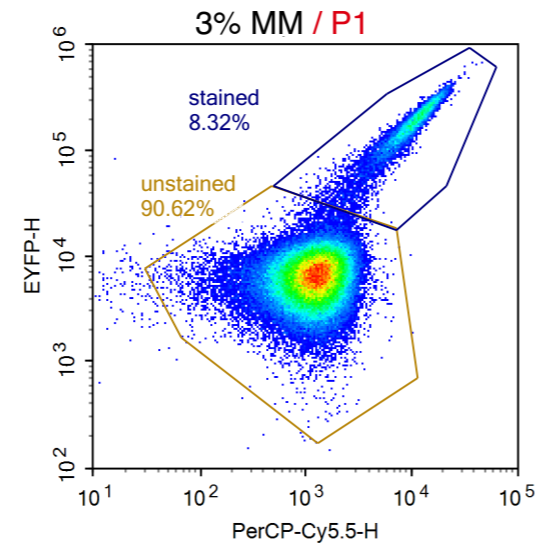**C**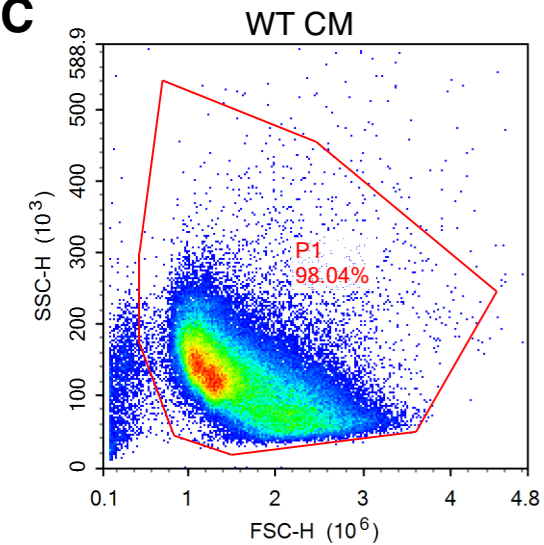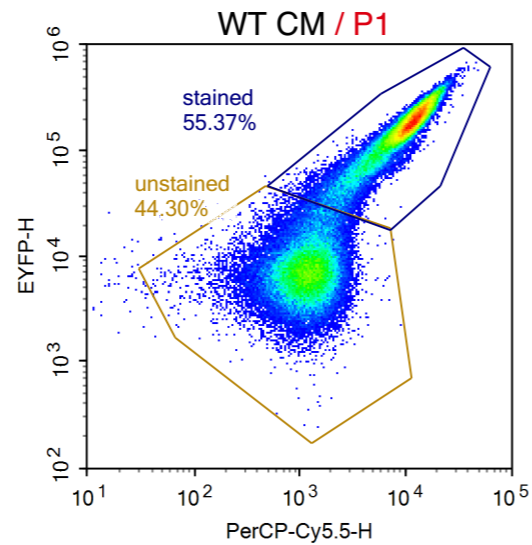**D**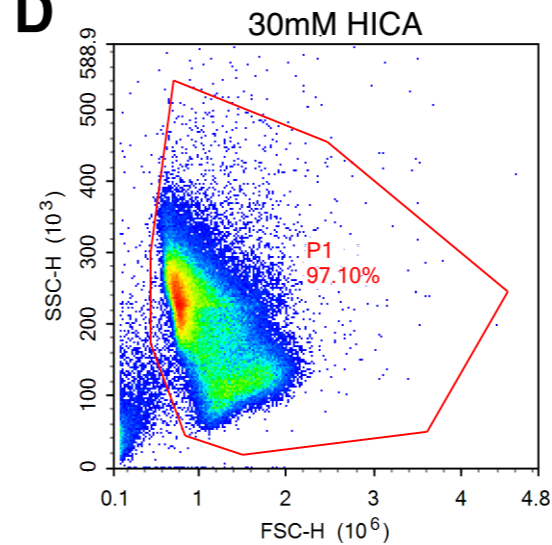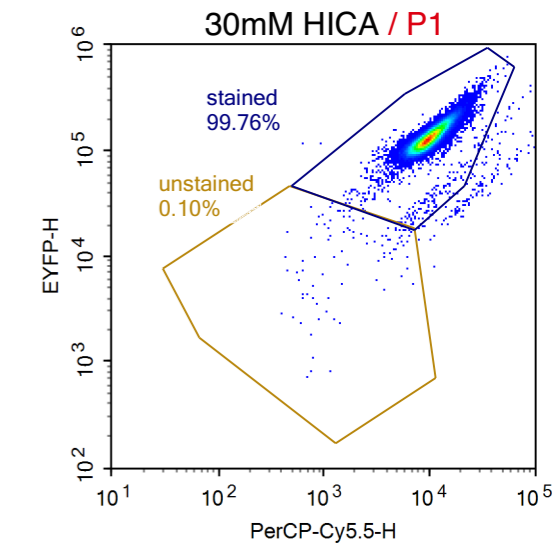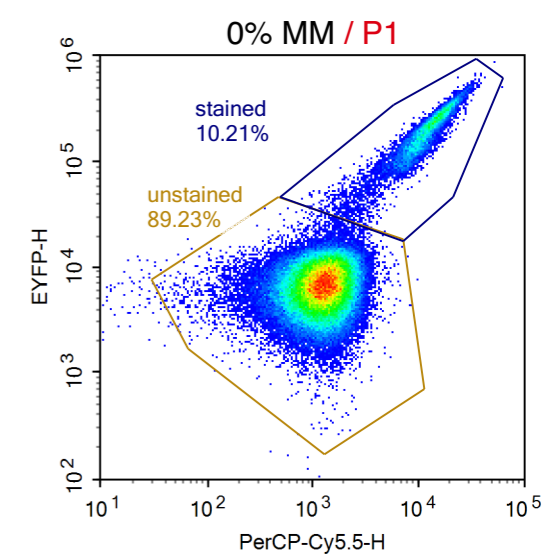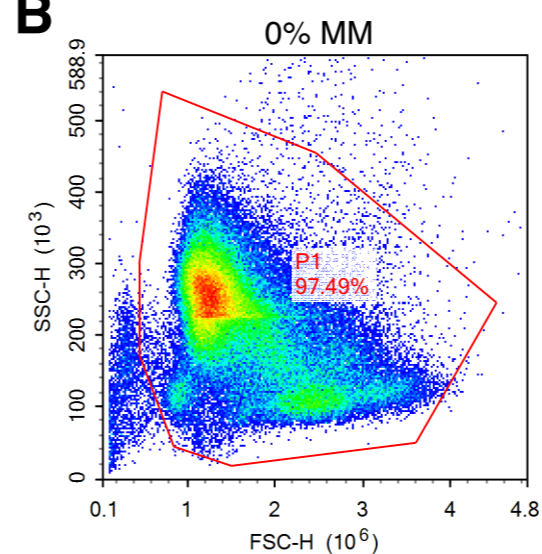

Supplement: S16 Fig — (A-D) Fission yeast cells were first gated on a FSC/SSC scatter plot as the red P1 gate (left panel). Then, cells gated on the blue and yellow gates were defined as phloxine B-stained dead cells and unstained living cells, respectively (right panel). The total count of each sample is 100,000 counts. Example images of flow cytometry data for cells in (A) 3% MM, (B) 0% MM, (C) WT CM, and (D) 30 mM HICA. (PDF) [file pbio.3001844.s016.pdf]

**A**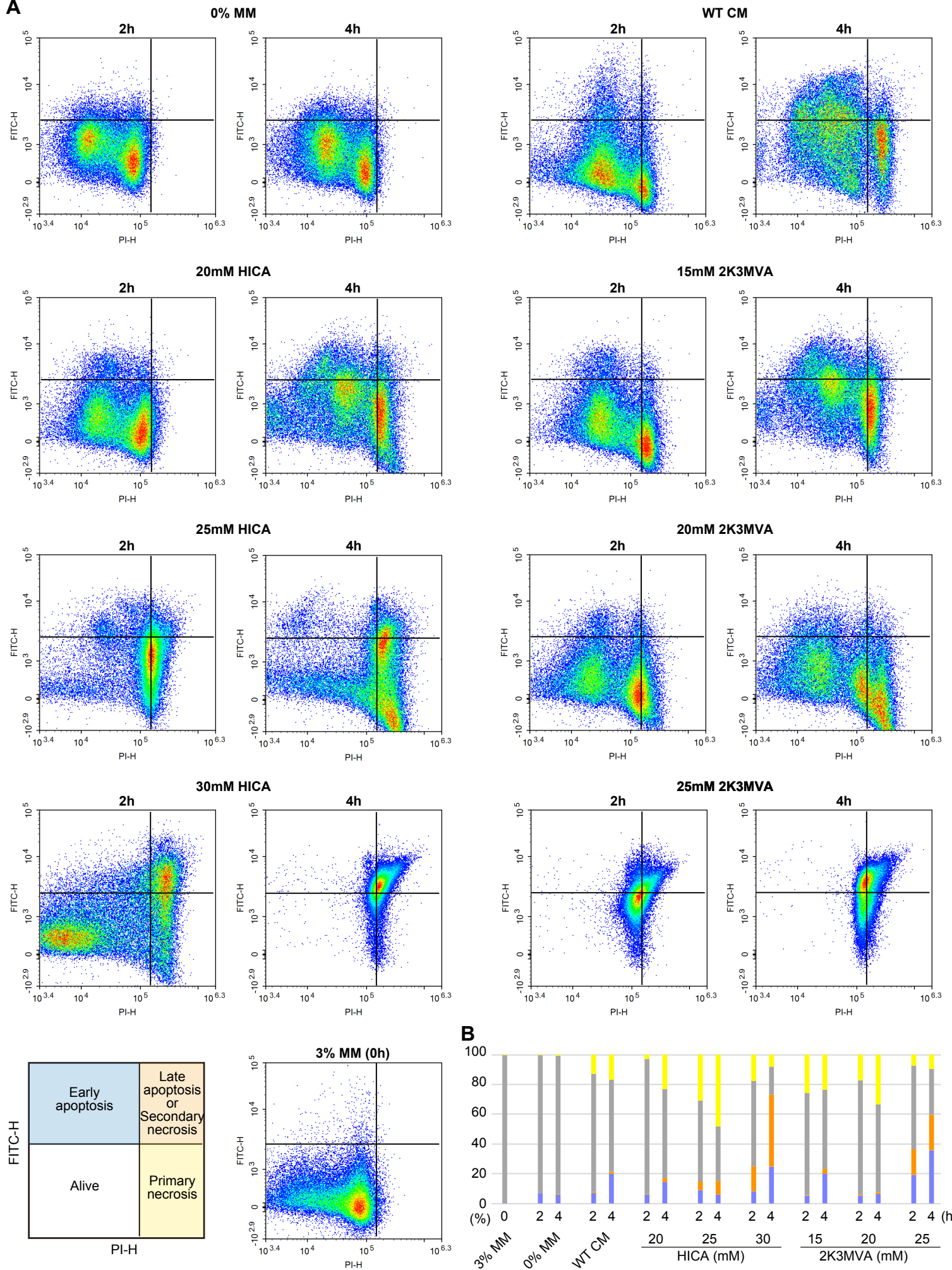

Supplement: S17 Fig — (A) AnnV/PI staining of WT cells in various media. Cells were inoculated to 0% MM, WT CM, 0% MM with 20/25/30 mM HICA, and 0% MM with 15/20/25 mM 2K3MVA from 3% MM (0h), and then stained at 2 h and 4 h after the inoculation. The bottom left panel shows the schematic image of the gate setting and their biological annotations for the panel (B). Early apoptotic cells exhibit phosphatidylserine externalization, which is detected by AnnV staining (AnnV+/PI−). Primary necrotic cells show a ruptured plasma membrane, which is detected by PI staining (AnnV−/PI+). Late apoptotic/secondary necrotic cells show both phosphatidylserine externalization and membrane permeability (AnnV+/PI+), while living cells are not stained with both AnnV and PI (AnnV−/PI−). B) Proportion of the cells in each phase. Percentages of cells in the early apoptosis (blue), in the primary necrosis (yellow), in the late apoptosis/secondary necrotic phase (orange), and alive cells (grey) are shown. The data underlying this figure can be found in S2 Data. (PDF) [file pbio.3001844.s017.pdf]

**A**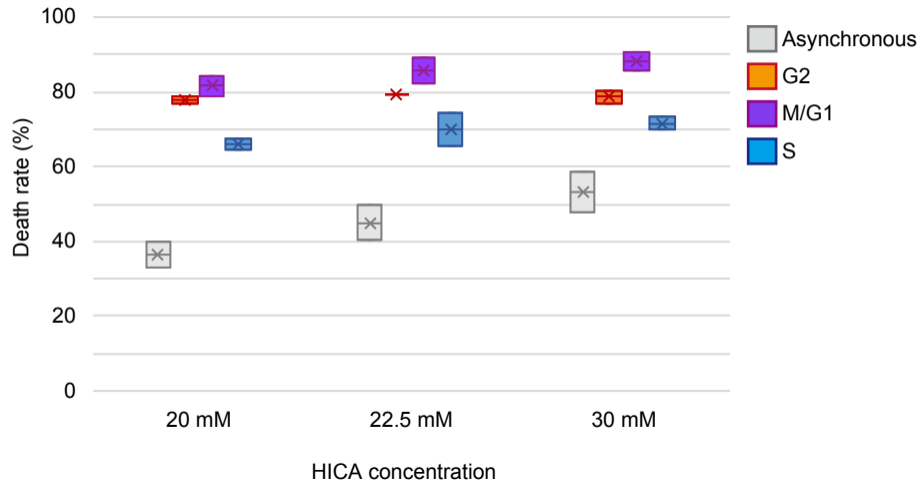**B**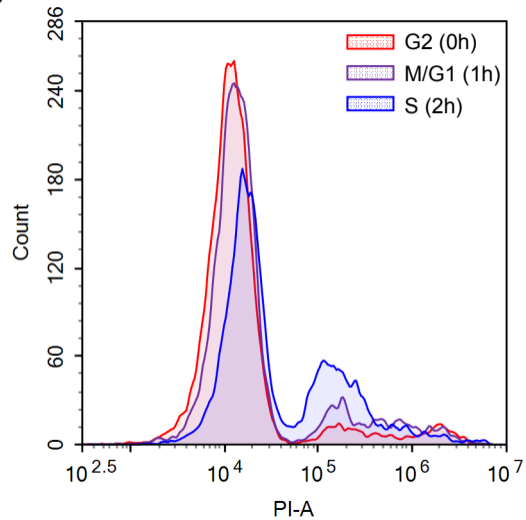

Supplement: S18 Fig — (A) Death rate of the cdc25-22 strain with and without a transient heat shock in 0% MM with HICA. G2, M/G1, and S cells were transferred to 0% MM with HICA 0, 1, and 2 h after a transient increase in temperature to 36°C. Death rate was measured after 8 h from media change. Asynchronous cells were transferred without a heat shock. The number of biological replicates is n = 2. (B) Flow cytometry analysis of synchronous cells. The first peak indicates the 1c cells, whose DNA amount is that of an interphase cell containing a G2 nucleus or a mitotic cell containing two G1 nuclei. The second peak indicates the 2c cells, whose DNA amount is that a septated cell containing 2 G2 nuclei. The ratio of 1c and 2c cells in each sample is given as: 87.8 ± 2.1%: 12.2 ± 2.1% for G2 cells, 76.0 ± 6.6%: 24.0 ± 6.6% for M/G1 cells, and 66.0 ± 1.7%: 34.0 ± 1.7% for S cells. The data underlying this figure can be found in S2 Data. (PDF) [file pbio.3001844.s018.pdf]

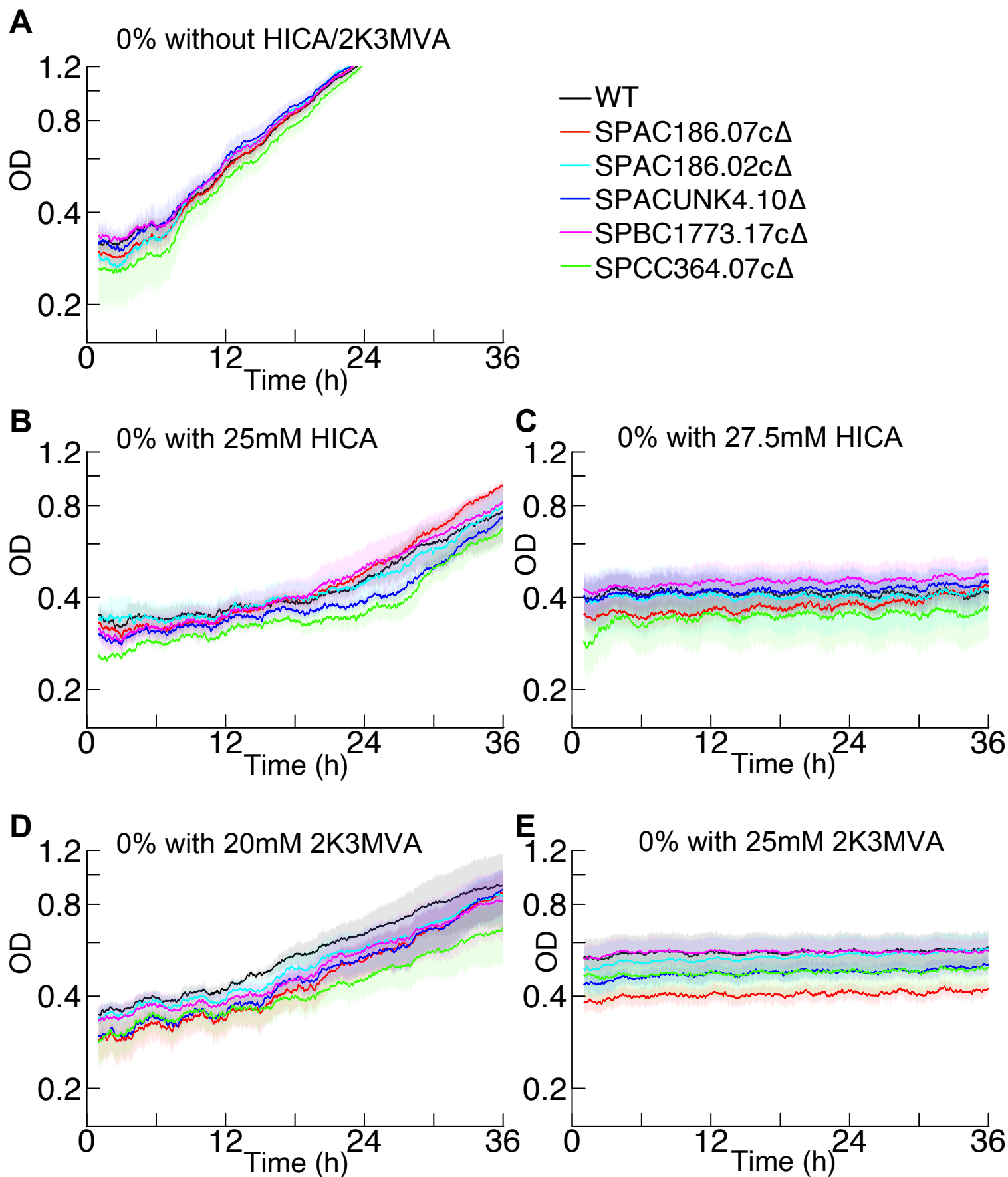

Supplement: S19 Fig — The strains in S12 Fig cells were precultured in 3% MM and then shifted to 0% MM, 0% MM with 25, 27.5 mM HICA or 20, 25 mM 2K3MVA. Each line represents an average of n = 2–4 samples. The data underlying this figure can be found in S2 Data. (PDF) [file pbio.3001844.s019.pdf]

**A**

3% MM 30mM HICA vs 3% MM

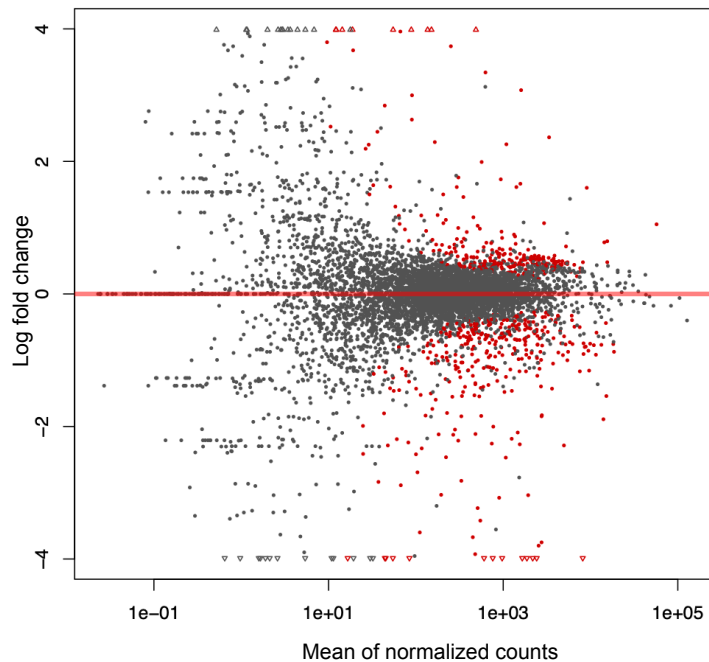**B**

3% MM 25mM 2K3MVA vs 3% MM

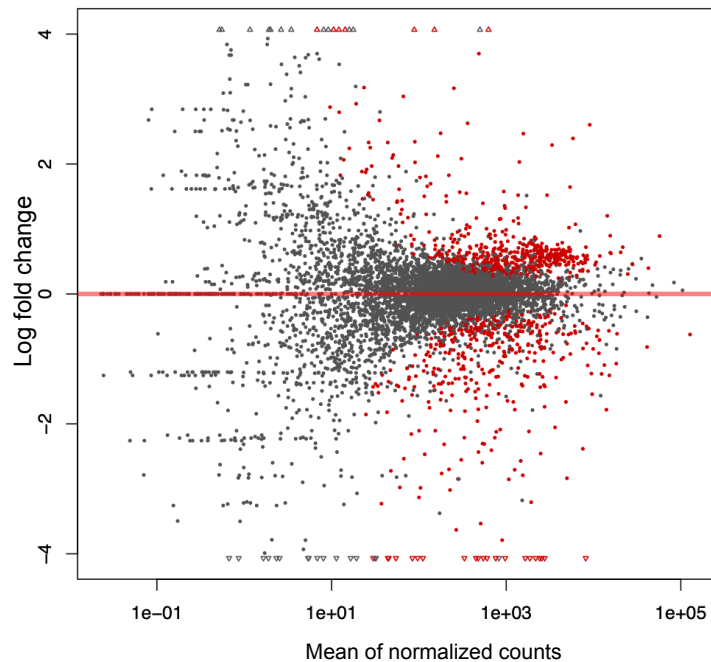

Supplement: S20 Fig — MA-plot for the log fold change of all genes between cells cultured in 3% MM with 30 mM HICA vs. those in 3%MM (A), and between cells cultured in 3% MM with 25 mM 2K3MVA vs. those in 3% MM (B). Cells precultured in 3% MM were cultured 3% MM with 30 mM HICA, 3% MM with 25 mM 2k3MVAm, and 3% MM for 24 h. Then, a difference in the gene expression was analyzed by RNA-seq. Red points indicate genes with a p-value less than 0.01. The data underlying this figure can be found in S7 Data. (PDF) [file pbio.3001844.s020.pdf]

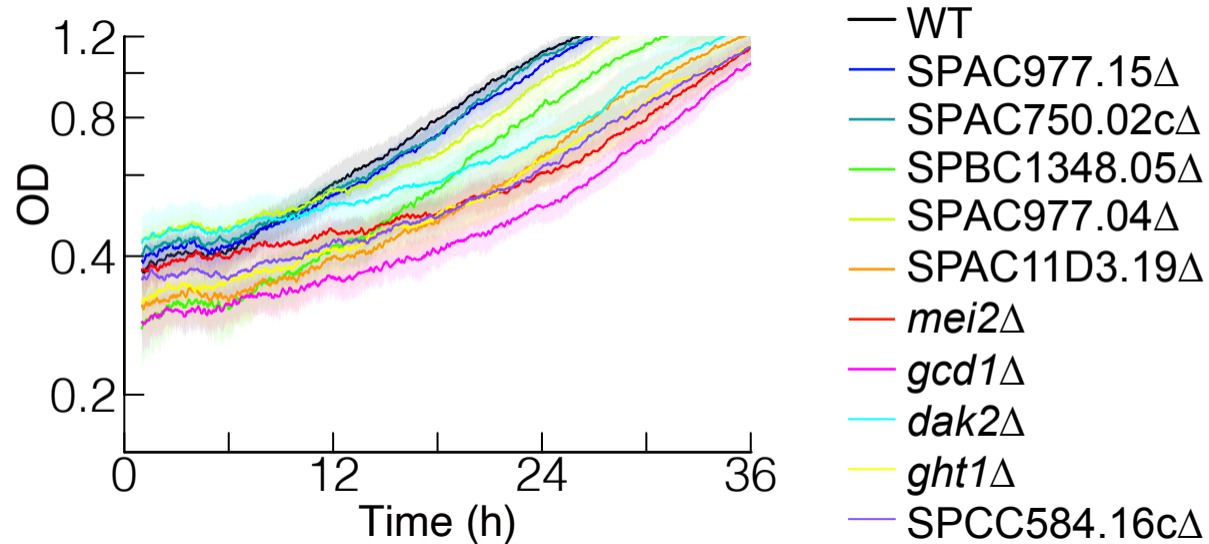

Supplement: S21 Fig — Each line represents an average of n≥4 samples. See Fig 3L for growth curves of the mutants that showed the significant prolongation of the delay phase longer than 30 h. The data underlying this figure can be found in S1 Data. (PDF) [file pbio.3001844.s021.pdf]

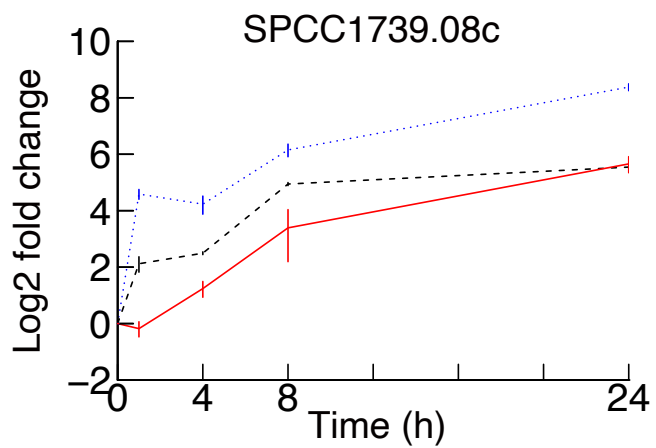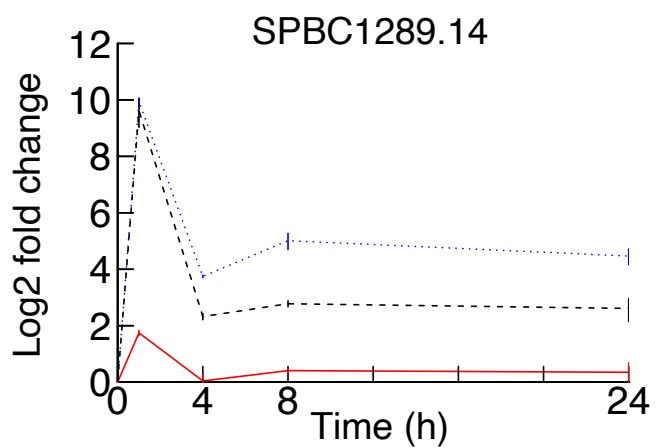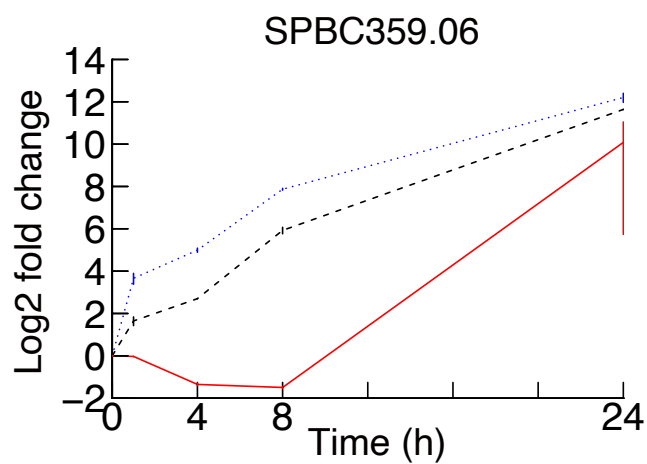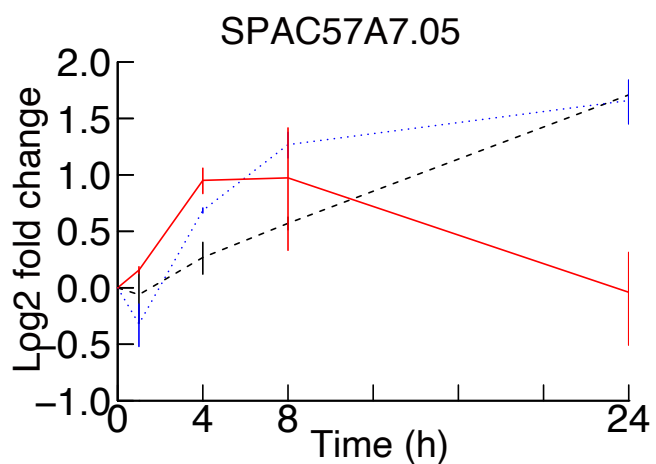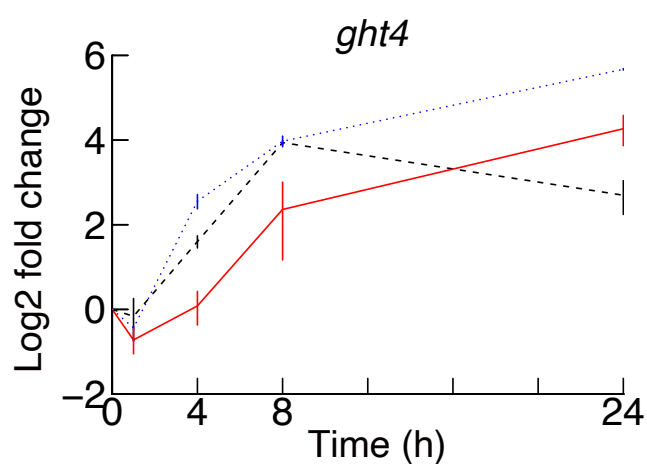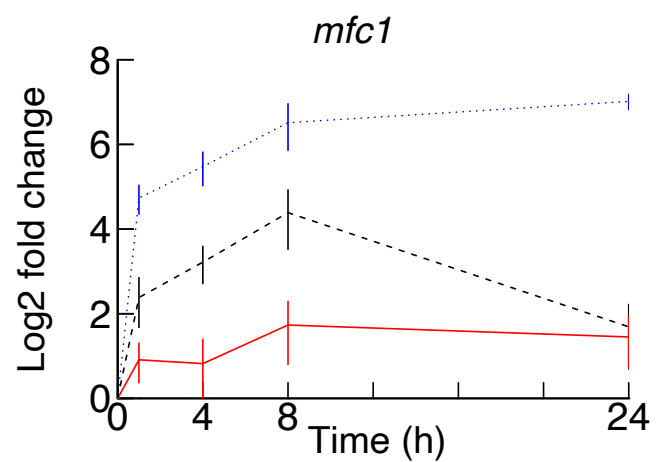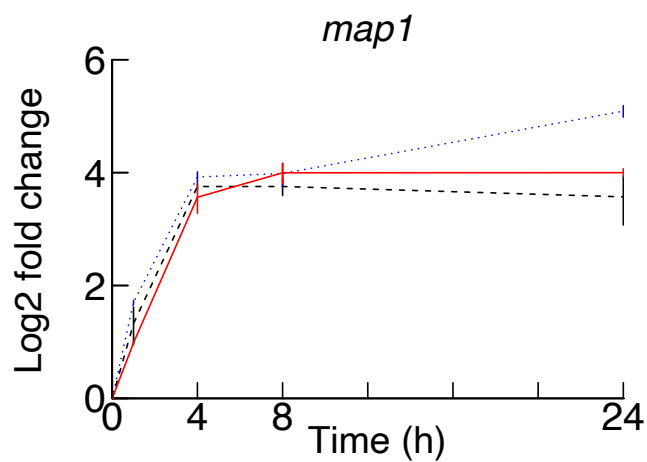

Supplement: S22 Fig — WT cells were precultured in 3% MM and inoculated to 3% MM with 30 mM HICA or 25 mM 2K3MVA and CM with 3% glucose. Expression of genes of which deletion mutants showed the significant prolongation of the delay phase, as shown in Fig 3L, was quantified by RT-PCR at times 0, 1, 4, 8, and 24 h after a change of media. Each line represents an average of n = 2 samples. The data underlying this figure can be found in S2 Data. (PDF) [file pbio.3001844.s022.pdf]

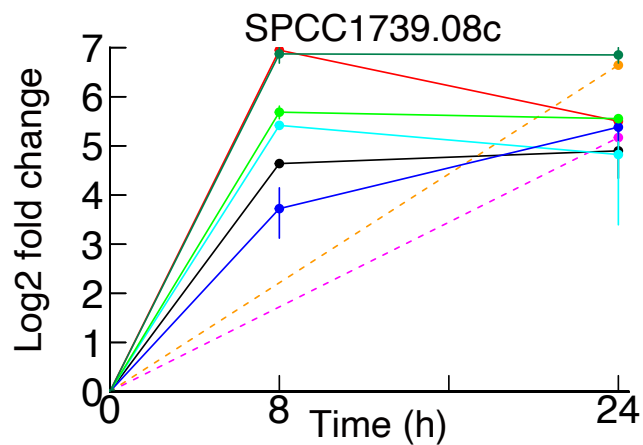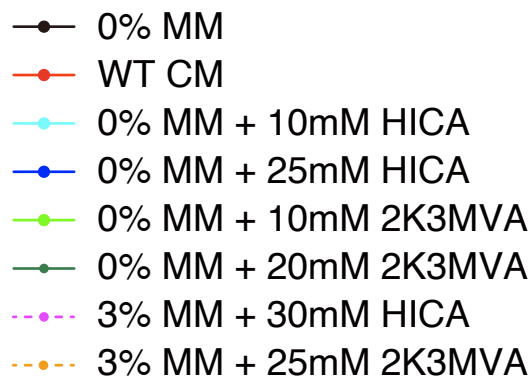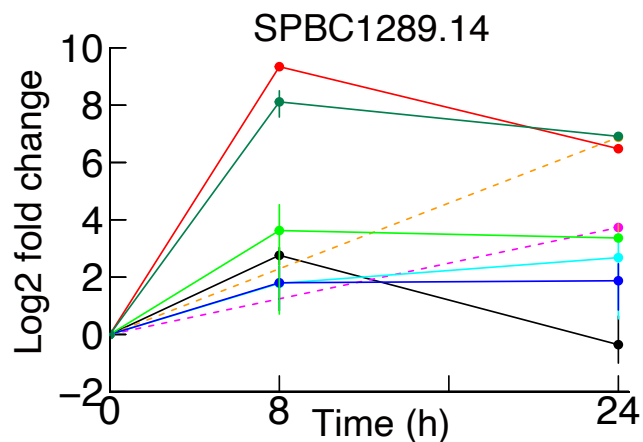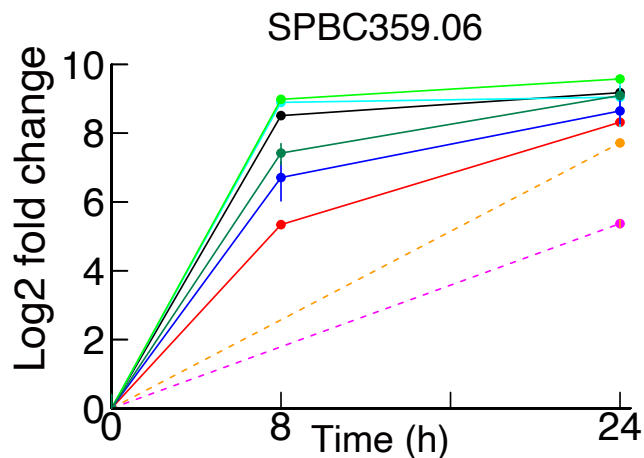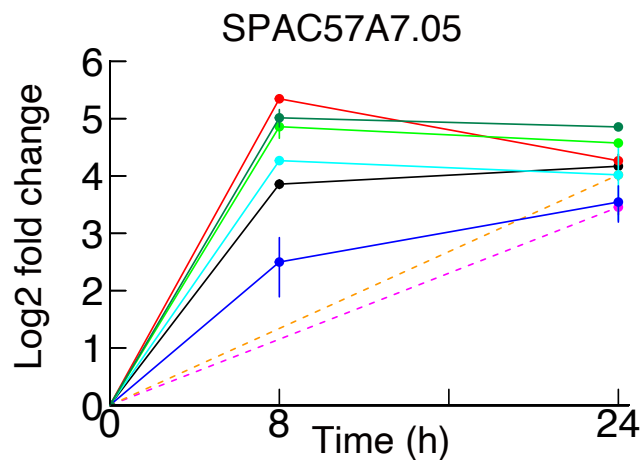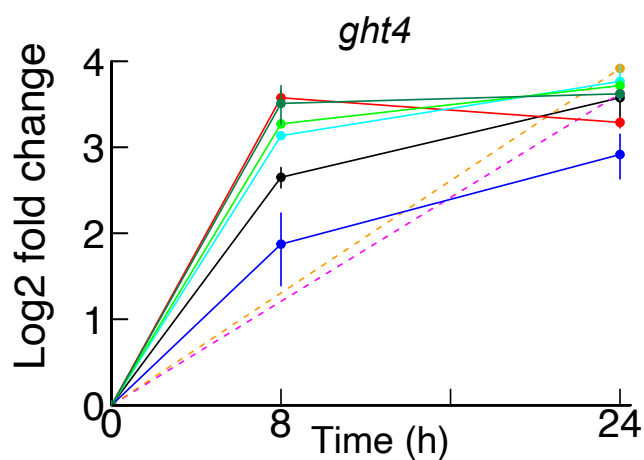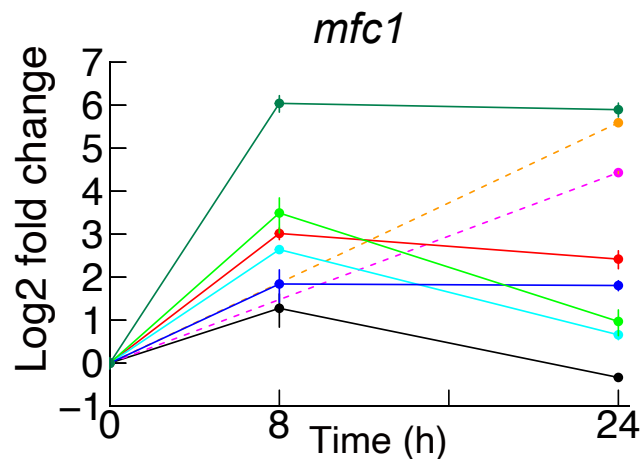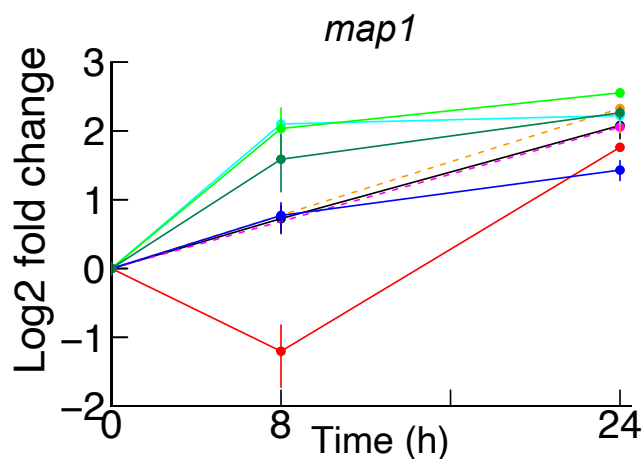

Supplement: S23 Fig — WT cells were precultured in 3% MM and inoculated to WT MC, 0% MM with or without 10, 25 mM HICA, 10, or 20 mM 2K3MVA, and 3% MM with 30 mM HICA or 25 mM 2K3MVA. Expression of genes of which deletion mutants showed the significant prolongation of the delay phase, as shown in Fig 3L, was quantified by RNAseq at times 0, 8, and 24 h after a change of media. Each line represents an average of n = 2 samples. The data underlying this figure can be found in S2 Data. (PDF) [file pbio.3001844.s023.pdf]

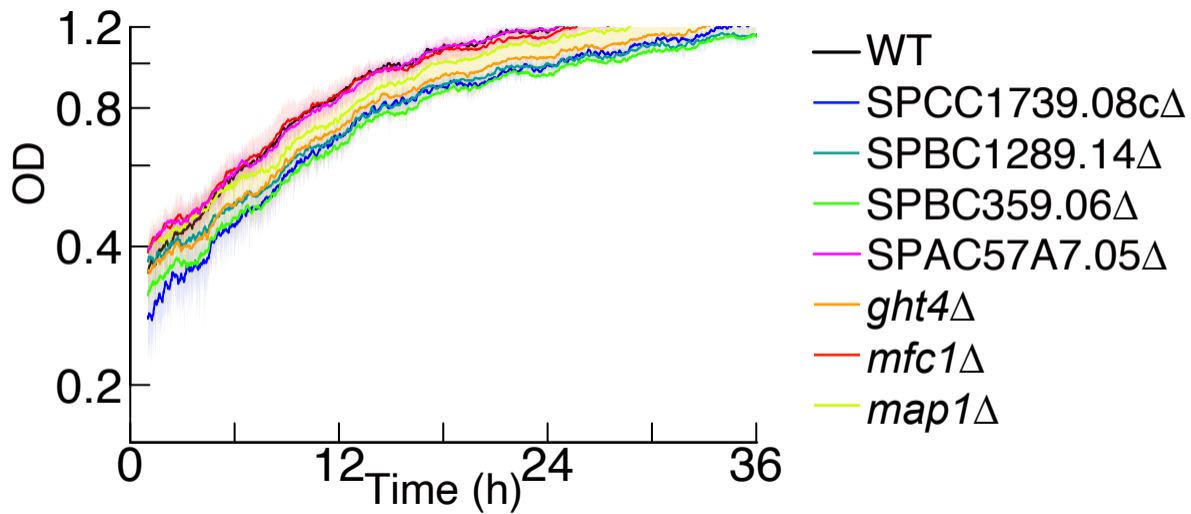

Supplement: S24 Fig — Mutant cells that showed the significant prolongation of the delay phase in Fig 3L were precultured in 0% MM, and their growth was measured in WT CM. Each line represents an average of n = 2 samples. The data underlying this figure can be found in S2 Data. (PDF) [file pbio.3001844.s024.pdf]

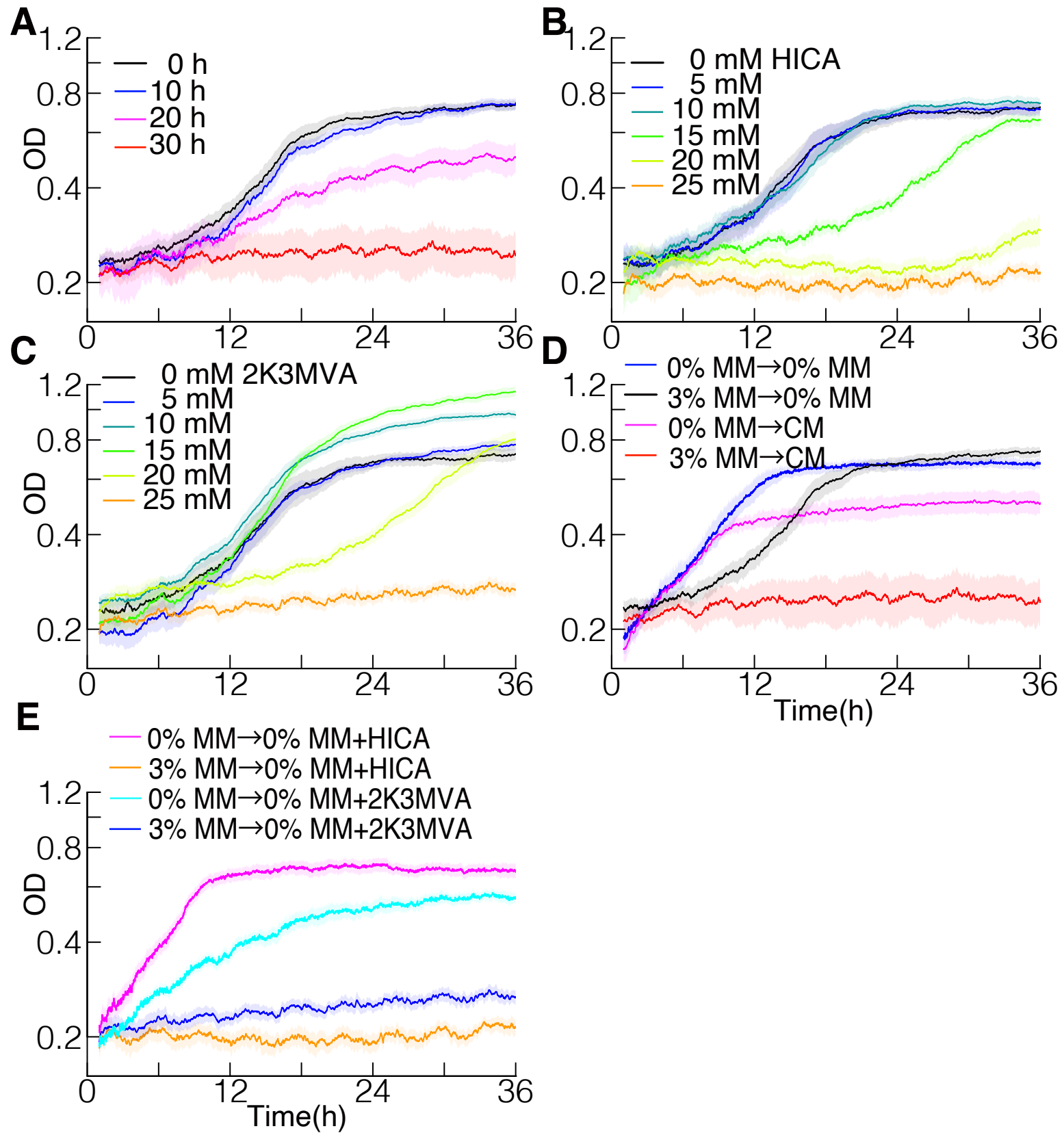

Supplement: S25 Fig — (A) Growth curves of 2 strains of S. cerevisiae (YEA8) in media conditioned by itself. Different colored lines indicate growth curves in CM with different incubation times. Each line represents an average of n≥6 samples. (B) Growth curves of YEA8 in 0% MM with various concentrations of HICA. Each line represents an average of n≥5 samples. (C) Growth curves of YEA8 in 0% MM with various concentrations of 2K3MVA. Each line represents an average of n≥5 samples. (D) Growth curves of YEA8 precultured in 0% MM or 3% MM in CM of OC-2. Each line represents an average of n≥6 samples. (E) Growth curves of YEA8 precultured in 0% MM or 3% MM in 0% MM with 25 mM HICA or 25 mM 2K3MVA. Each line represents an average of n≥6 samples. The data underlying this figure can be found in S2 Data. (PDF) [file pbio.3001844.s025.pdf]

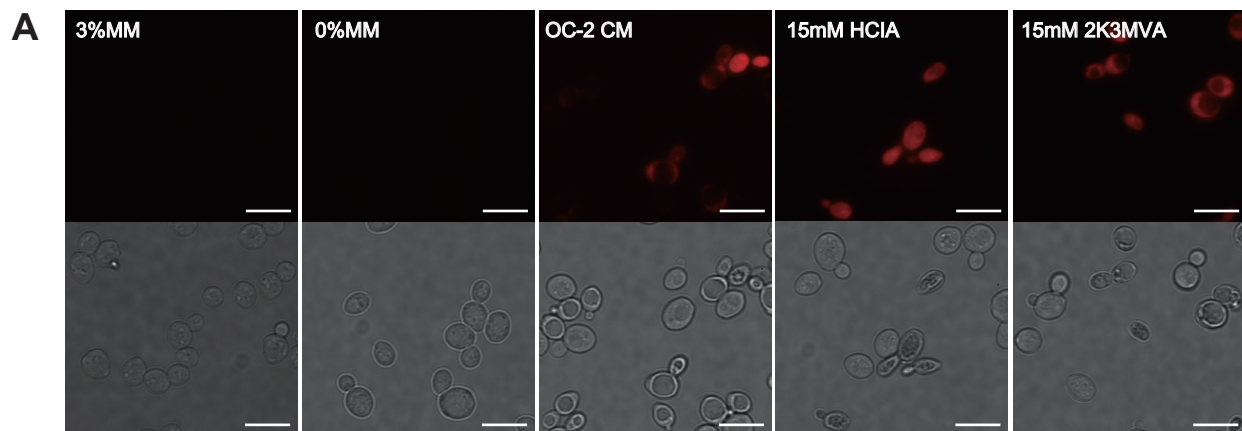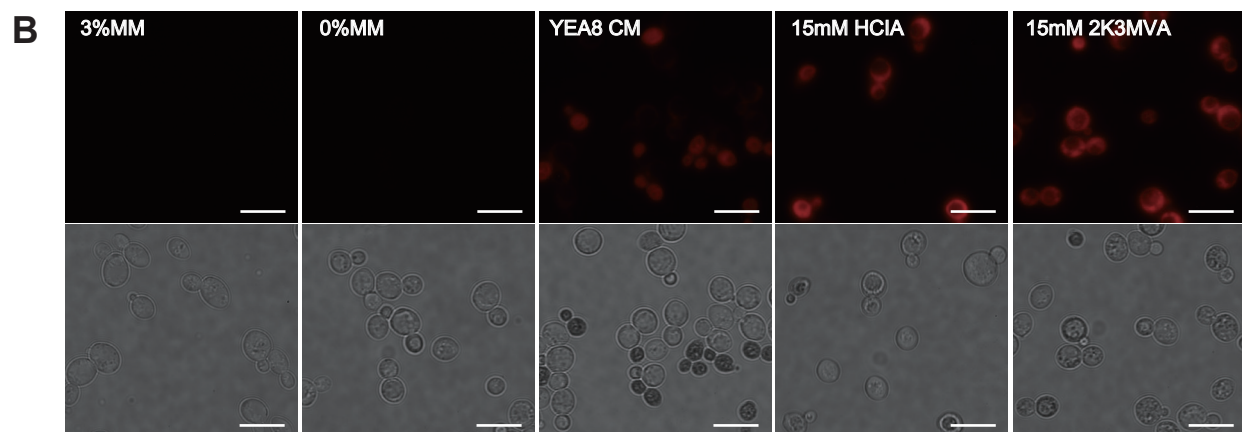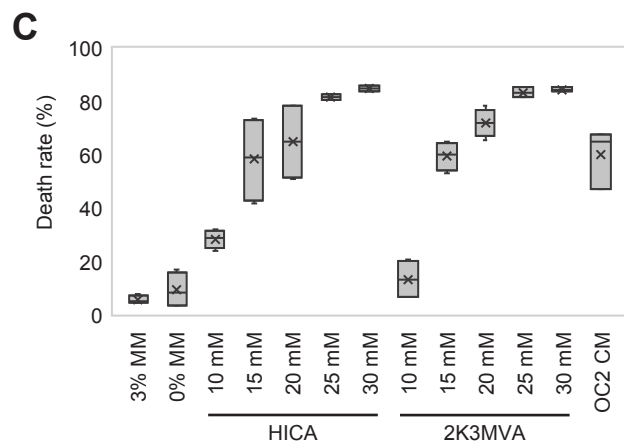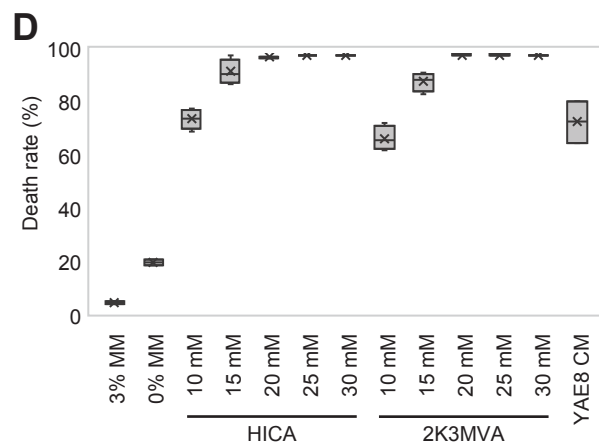

Supplement: S26 Fig — (A and B) Fluorescent (upper) and brightfield (bottom) microscopic images of WT S. cerevisiae strains (A) OC2, (B) YEA8 in various media after 8 h of incubation. Cells precultured in 3% MM were transferred to 3% MM, 0% MM, CM of each strain, 0% MM with 15 mM HICA, and 15 mM 2K3MVA. In fluorescent microscopic images, dead cells were stained with phloxine B. Scale bar indicates 10 μm. (C and D) Box plot of the phloxine B stained cell ratio after 8 h of incubation. S. cerevisiae (C) OC2, (D) YEA8 cells were precultured in 3% MM and shifted to various media. Grey areas represent the interquartile ranges of dyed cell ratio in each sample, and crosses represent the mean value. Fluorescence of over 50,000 cells were measured for each sample using FACS. (n = 2–4) The data underlying this figure can be found in S2, S4, and S5 Data. (PDF) [file pbio.3001844.s026.pdf]
