# Supplementary material for: Autotoxin-mediated latecomer killing in yeast communities
Source: PLoS Biol. 2022 Nov 7;20(11):e3001844. doi: 10.1371/journal.pbio.3001844 (PMC9639812; doi:10.1371/journal.pbio.3001844)
Supplement: S4 Fig — (A) Growth curves of WT cells in the WT CM with 3% glucose, 3% fructose, 3% galactose, 3% mannose, or 3% 2-deoxy-D-glucose (2-DG). Each line represents an average of n = 4–6 samples. (B) Growth curves of WT cells in the WT CM with 3.75 g/L (22.2 mM) monosodium glutamate or an amino acid mix. The final amino acid concentration in amino acid mix sample was as follows: Adenine 10 mg/L, L-Arginine HCl 50 mg/L, L-Aspartic Acid 80 mg/L, L-Histidine HCl 20 mg/L, L-Isoleucine 50 mg/L, L-Lysine HCl 50 mg/L, L-Methionine 20 mg/L, L-Phenylalanine 50 mg/L, L-Threonine 100 mg/L, L-Tryptophan 50 mg/L, Uracil 20 mg/L, L-Tyrosine 50 mg/L, L-Valine 140 mg/L. Each line represents an average of n = 2–4 samples. The data underlying this figure can be found in S2 Data. (PDF) [file pbio.3001844.s004.pdf]

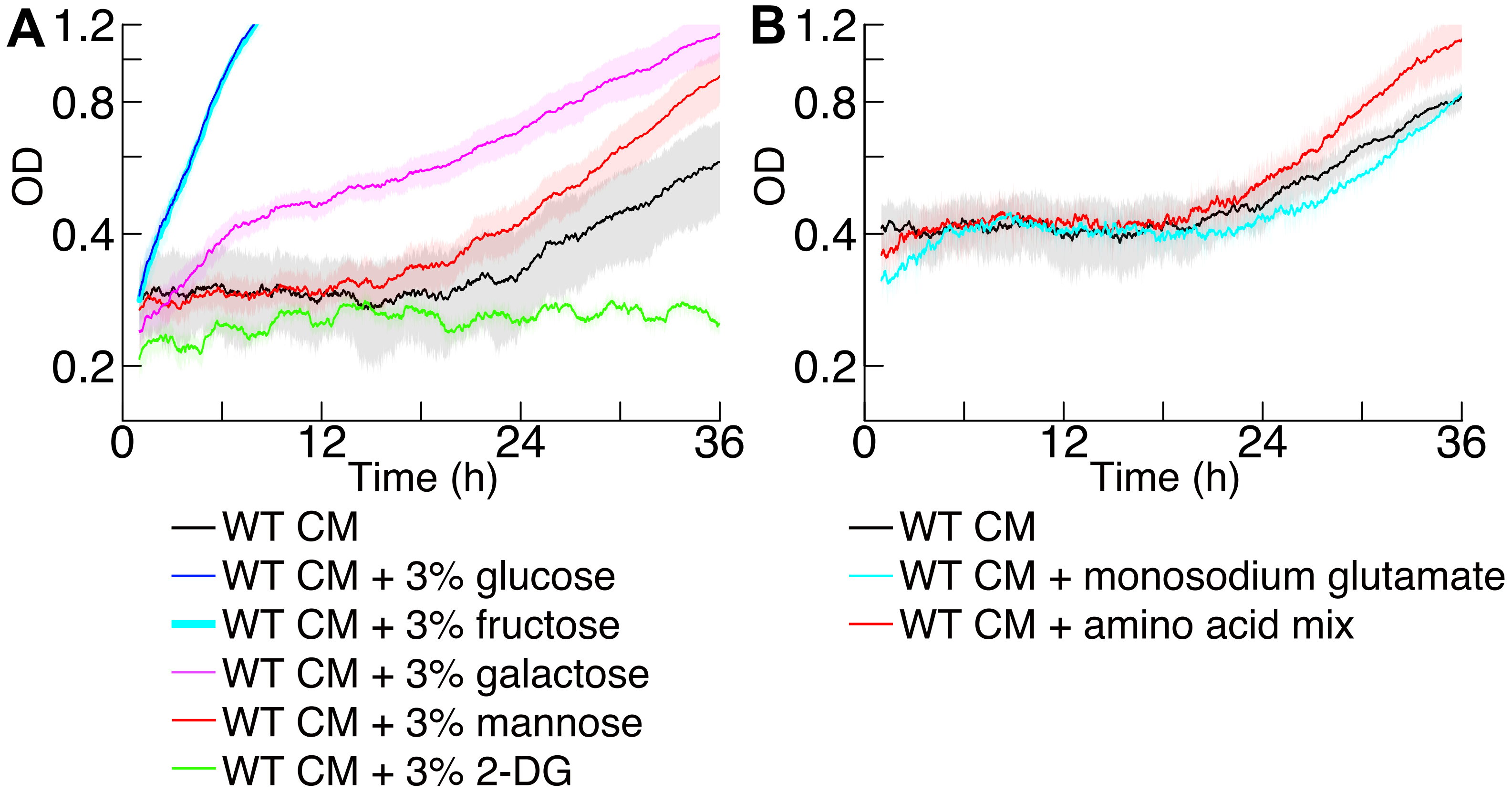
A
B
OD
1.2
0.8
0.4
0.2
0
12
24
36
Time (h)
WT CM
WT CM + 3% glucose
WT CM + 3% fructose
WT CM + 3% galactose
WT CM + 3% mannose
WT CM + 3% 2-DG
WT CM
WT CM + monosodium glutamate
WT CM + amino acid mix
