# Supplementary material for: Autotoxin-mediated latecomer killing in yeast communities
Source: PLoS Biol. 2022 Nov 7;20(11):e3001844. doi: 10.1371/journal.pbio.3001844 (PMC9639812; doi:10.1371/journal.pbio.3001844)
Supplement: S6 Fig — The common mutations detected in all 4 samples are shown in yellow. Mutations detected in both original WT and surviving cells, but not in all 4, are shown in red. Unique mutations detected only in a certain replicate of the original WT, and surviving cells are shown in blue and cyan, respectively. The data underlying this figure can be found in S6 Data. (PDF) [file pbio.3001844.s006.pdf]

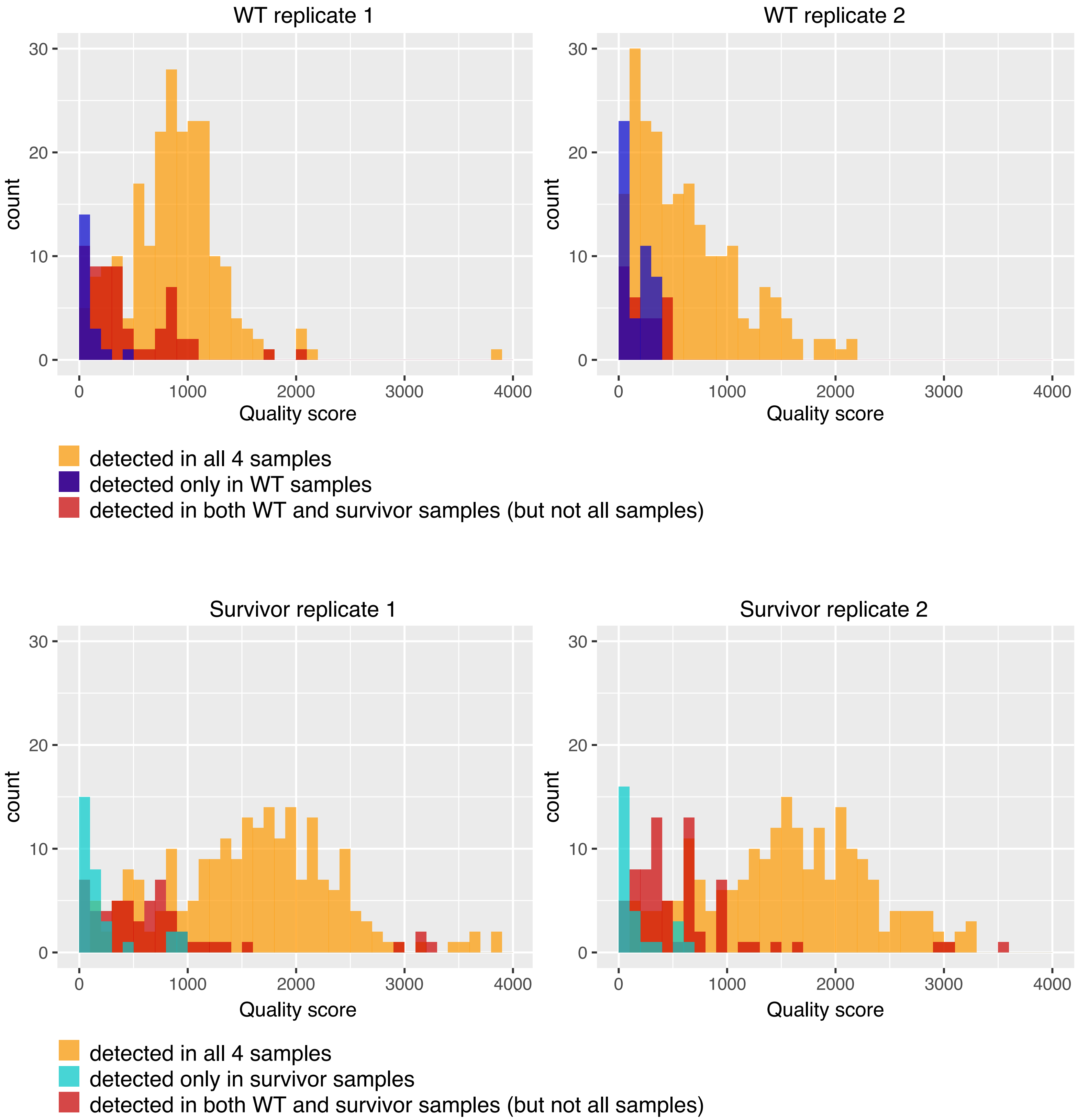

WT replicate 1
WT replicate 2
Survivor replicate 1
Survivor replicate 2
count
Quality score
0
10
20
30
0
1000
2000
3000
4000
detected in all 4 samples
detected only in WT samples
detected in both WT and survivor samples (but not all samples)
detected in all 4 samples
detected only in survivor samples
detected in both WT and survivor samples (but not all samples)
