# Supplementary material for: Autotoxin-mediated latecomer killing in yeast communities
Source: PLoS Biol. 2022 Nov 7;20(11):e3001844. doi: 10.1371/journal.pbio.3001844 (PMC9639812; doi:10.1371/journal.pbio.3001844)
Supplement: S10 Fig — (A and B) Structures of (A) L- and (B) D-forms of HICA. (C and D) Growth curves of WT cells in 0% MM with various concentrations of (C) L-form HICA and (D) D-form HICA. For the L-form of HICA, growth curves are an average of 15–18 samples, and for the D-form of HICA, it is an average of 2 samples. The data underlying this figure can be found in S2 Data. (PDF) [file pbio.3001844.s010.pdf]

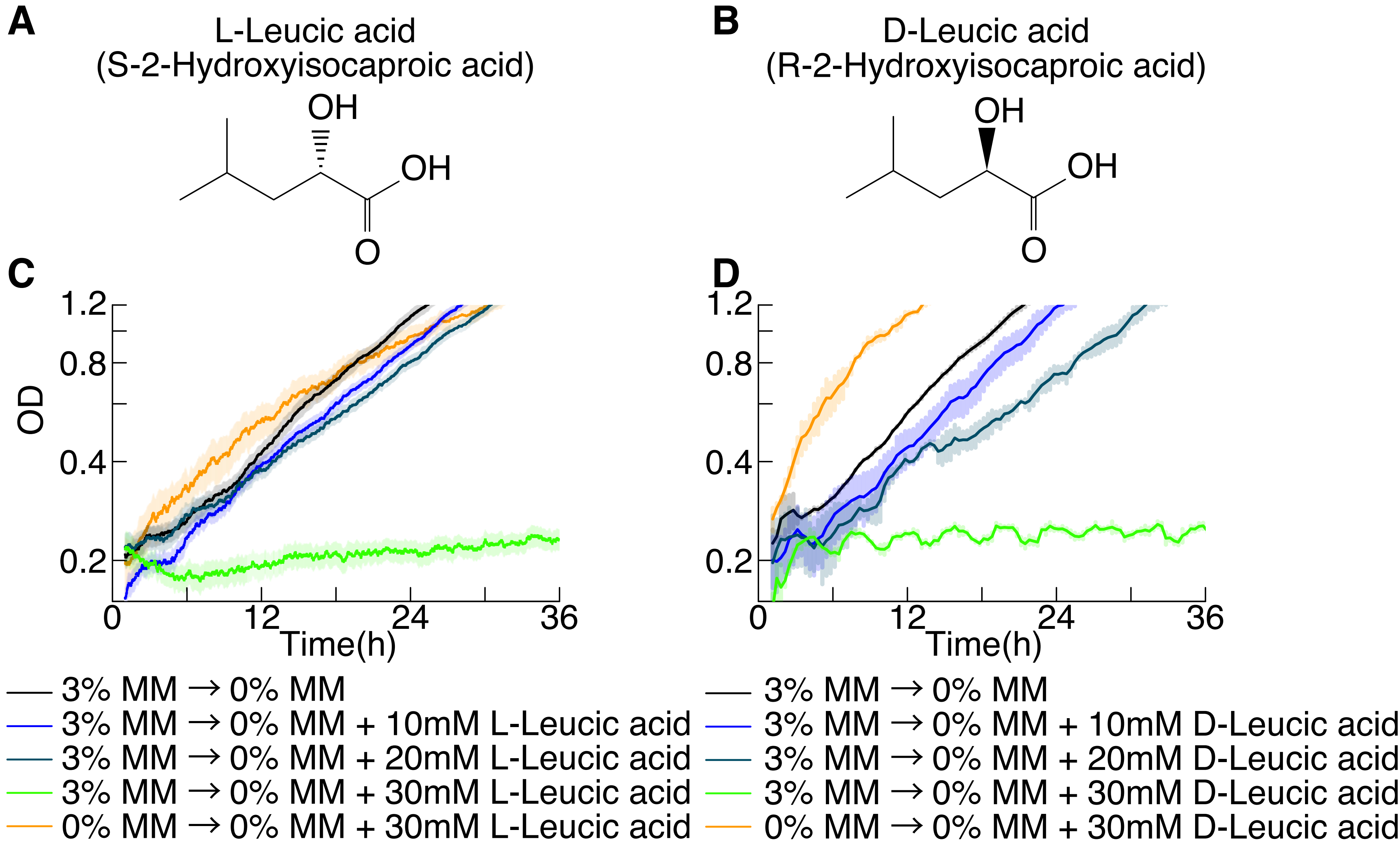
A
L-Leucic acid
(S-2-Hydroxyisocaproic acid)
OH
OH
O
B
D-Leucic acid
(R-2-Hydroxyisocaproic acid)
OH
OH
O
C
D
OD
1.2
0.8
0.4
0.2
0
12
24
36
Time(h)
3% MM → 0% MM
3% MM → 0% MM + 10mM L-Leucic acid
3% MM → 0% MM + 20mM L-Leucic acid
3% MM → 0% MM + 30mM L-Leucic acid
0% MM → 0% MM + 30mM L-Leucic acid
3% MM → 0% MM
3% MM → 0% MM + 10mM D-Leucic acid
3% MM → 0% MM + 20mM D-Leucic acid
3% MM → 0% MM + 30mM D-Leucic acid
0% MM → 0% MM + 30mM D-Leucic acid
