# Supplementary material for: Autotoxin-mediated latecomer killing in yeast communities
Source: PLoS Biol. 2022 Nov 7;20(11):e3001844. doi: 10.1371/journal.pbio.3001844 (PMC9639812; doi:10.1371/journal.pbio.3001844)
Supplement: S1 Text — Text A-D and Table A-G. (PDF) [file pbio.3001844.s027.pdf]

Supporting Materials for “Autotoxin-mediated latecomer killing in yeast communities.”

Arisa H. Oda, Miki Tamura, Kunihiko Kaneko, Kunihiro Ohta, and Tetsuhiro S. Hatakeyama

Supporting Texts

Supporting Text A: Definition of the length of delay phase and growth rate

In general, when we transfer cells from a glucose-rich medium to a glucose-depleted medium, cells show a lag phase where they switch intracellular activities to adapt to the new environment before starting growth. If we transferred cells to conditioned media and media with inhibitory molecules, a delay phase was observed in addition to the lag phase. We defined the length of lag and delay phases as τ. We assumed that the lag and delay phases were linearly separable, and thus a difference between τ and τ_0, which was τ for 0% MM, would give the length of the delay phase. Here, τ was measured as the time when the OD reached $2a_0$, where a_0 is the OD at the initial state. It was measured as a_0 as the average OD from 1–2 h because OD fluctuates in time, especially during the 1st hour, owing to the apparatus.

In addition, we defined the steady-state growth rate of cells after the lag and delay phases as r (see also S14 Fig for the definition of τ, a_0, and r). r was measured within different ranges in different media due to variations in the delay phase length; 20–25 h for 0% MM, 25–35 h for WT CM, from 35–50 h for *fbp1*Δ CM, 15–30 h for 20 mM HIAC, 22.5 mM HICA, and 17.5 mM 2K3MVA, 30–45 h for 25 mM HICA, 25–40 h for 20 mM 2K3MVA, and 30–60 h for 22.5 mM 2K3MVA.

See Table C for the measured τ, r, and a_0.

Supporting Text B: Identification of mutated sites in surviving cells in conditioned media

We obtained 1.2×10^6 read pairs or more for each sample. After the removal of low-quality reads (phred quality $\geq$ Q15 and $>$ 10 base limit) using fastp (an ultra-fast all-in-one FASTQ pre-processor; version 0.20.0, (1)), we aligned the Illmina short reads to the reference genome of *Schizosaccharomyces pombe* (version 2018.09.04 (2)) on the PomBase database ((3)) using BWA (version 0.7.7, (4)). The average coverage depth for the reference chromosomes was 90× or more for each sample. We analysed mutations in each aligned dataset using the Genome Analysis Tool Kit-HaplotypeCaller software (GATK; version 4.1.4.1, (5, 6)) with the default parameters with an option of ‘ploidy=1’. Then, we refined the raw variants identified by HaplotypeCaller using Variant filtration, with parameters ‘QD < 2.0 or FS > 60.0, MQ < 40.0, or MQRankSum < -12.5 or ReadPosRankSum < -8.0 or SOR > 4.0’, as recommended. We identified 271 and 268 SNPs and InDels, respectively, in two replicates of WT samples. Also 285 and 292 SNPs and InDels, respectively in the surviving samples. We searched for “common” and “unique” mutations in each sample using vcftools (version 0.1.13, (7)).

We classified each mutation into three groups: 1) Mutations that are common in all four samples; 2) mutations detected in both original WT and survivor samples but not in all samples; 3) mutations detected uniquely in a particular sample. We plotted the quality scores of mutations in each group as histograms in S6 Fig.

Quality scores of mutations commonly found in all four samples (yellow bars in S6 Fig) were relatively high, and such mutations in the group 1 were expected to be the original SNPs and InDels in WT and be not the result of culturing in WT CM.

Mutations in group 2 tended to show lower quality scores than those in group 1 (see red bars in S6 Fig). Mutations that were detected in only one of the survivor samples or both WT replicates, and one of the two survivor samples might have resulted from culturing in WT CM. Therefore, these 35 mutations were checked, and mutations were false positives.

Mutations in group 3 were unique mutations found in one sample (see blue bars for WT and cyan bars for surviving cells in S6 Fig). To check whether these mutations arose during the culture in WT CM or not, we analysed all 84 unique mutations in the surviving samples. These unique mutations were located at repetitive sequences around telomeres and centromeres, where false positives are often found (8). In addition, the quality scores for these mutations were the lowest among the three groups; therefore, they were false positives.

Supporting Text C: Estimation of the steady-state ratio of cells in the competition assay

In the competition assay, adapted cells started to grow earlier than unadapted cells because they showed neither the lag nor the delay phase. Later, unadapted cells adapted to toxins, and their growth rate was the same as adapted cells. Hence, the ratio of adapted to unadapted cells reached a constant value at the steady-state. We calculated the steady-state ratio of cells as follows:

The adapted cell growth rate was r during τ hours, while the concentration of unadapted cells doubled after τ hours. Hence, if we assumed that the ratio of cells reached the steady state immediately after τ hours, the steady-state fraction of the adapted cells would be given as

$$\frac{\exp(r\tau)}{\exp(r\tau)+2} = \frac{\exp(0.056 \times 32.77)}{\exp(0.056 \times 32.77)+2} = 0.758. \tag{1}$$

This estimated value agreed well with the measured values obtained in the competition assay (see Fig 2 in the main text).

Supporting Text D: Estimation of the death rate from growth rate and the length of delay phase

We assumed that cells were divided into two types: dead cells, which did not grow, and living cells, which showed exponential growth. The summation of both cells was observed as OD (see S14 Fig). Then, the growth curve was drawn as follows:

$$\mathrm{OD}(t) = da_0 + (1-d)a_0 \exp(rt), \tag{2}$$

where d is the death rate at time $= 0$. Thus, d is calculated from r and τ as follows:

$$d = \frac{2-\exp(r\tau)}{1-\exp(r\tau)}, \tag{3}$$

We estimated the death rate for each sample. Estimated death rates were well correlated with flow cytometry results (see S15 Fig).

In 0% MM and CM, the estimated values were slightly higher than the measured values. In Eq. 2, we ignored the lag phase of living cells, which might cause an overestimation of the death rate. Indeed, the difference between the estimated and measured death rates in CM as almost the same as that in 0% MM.

In addition, at a high concentration of 2K3MVA, death rates were underestimated. In Eq. 2, we assumed that the growth rate was independent of time. If the growth rate decreased with time; that is, the concentration of living cells was given as a convex function, the death rate would be underestimated. In media with a high concentration of 2K3MVA, r was much lower than that in other media (see Table C). This suggested that administration of 2K3MVA at a high concentration decreased the growth rate over time. Then, the death rate was underestimated. In any case, the delay phase was mainly caused by cell death.

Supporting Tables

Table A. List of compounds detected from a minimal medium and conditioned media
(Abbreviations: N.D., Not detected; N.R., Not reproduced)

					S. pombe		*S. cereviciae*	
Compound name	Pubchem ID	HMDB ID	m/z	0% MM	WT CM	*fbp1Δ CM*	OC-2 CM	YEA8 CM
2-Hydroxy-4-methylvaleric acid (HICA)	439960	HMDB00624	131.071	N.D.	+	+	+	+
3-Methyl-2-oxovaleric acid (2K3MVA)	47	HMDB0000491	129.055	N.D.	+	+	+	+
5-Oxoproline	7405	HMDB00267	128.035	N.D.	+	+	+	N.D.
2-Hydroxyglutaric acid	43	HMDB00606,HMDB00694	147.029	N.D.	+	+	+	N.D.
Ala	602	HMDB00161,HMDB01310	90.055	N.D.	+	+	+	+
Gln	738	HMDB00641,HMDB03423	147.076	N.D.	+	+	+	+
Glu	611	HMDB00148,HMDB03339	148.060	N.D.	+	+	+	+
Glycerol 3-phosphate (G3P)	439162	HMDB00126	171.006	N.D.	+	+	+	N.D.
Hypoxanthine	790	HMDB00157	137.045	N.D.	+	+	+	+
Inosine	6021	HMDB00195	269.086	N.D.	+	+	+	+
Phe	994	HMDB00159	166.085	N.D.	+	+	+	+
Succinic acid	1110	HMDB00254	117.019	N.D.	+	+	+	+
1-Methyladenosine	27476	HMDB03331	282.122	N.D.	+	N.R.	+	+
2-Hydroxyvaleric acid	98009	HMDB01863	117.055	N.D.	+	N.R.	+	+
Adenosine	60961	HMDB00050	268.104	N.D.	+	N.R.	N.D.	+
Citric acid	311	HMDB00094	191.019	N.D.	+	N.R.	+	+
Guanosine	6802	HMDB00133	284.099	N.D.	+	N.R.	+	+
Lactic acid	612	HMDB00190,HMDB01311	89.024	N.D.	+	N.D.	N.D.	N.D.
Pyruvic acid	1060	HMDB00243	87.009	N.D.	+	N.D.	N.D.	N.D.
Tyr	1153	HMDB00158	182.079	N.D.	+	N.R.	+	+
Terephthalic acid	7489	HMDB02428	165.019	N.R.	+	+	+	+
Adenine	190	HMDB00034	136.061	+	+	+	+	+
Glycerol	753	HMDB00131	93.055	+	+	+	+	+
His	773	HMDB00177	156.077	+	+	+	+	+
Leu	857	HMDB00687	132.102	+	+	+	+	+
Pantothenic acid	6613	HMDB00210	218.104	+	+	+	+	+
Uracil	1174	HMDB00300	113.034	+	+	+	+	+
Nicotinic acid	938	HMDB01488	124.039	+	+	+	+	+
Arg	6322	HMDB00517,HMDB03416	175.119	N.D.	N.R	+	N.D.	+
Gly	750	HMDB00123	76.040	N.D.	N.R	+	N.D.	N.D.
Glycerophosphocholine (GPCho)	439285	HMDB00086	258.109	N.D.	N.R	+	N.D.	N.D.
Lys	866	HMDB00182,HMDB03405	147.112	N.D.	N.R	+	N.D.	N.D.
Malic acid	525	HMDB00156,HMDB00744	133.015	N.D.	N.R	+	N.D.	+

Table B. HICA and 2K3MVA concentration analyzed by CE-MS and LC-MS/MS

Media	HICA (μM)	2K3MVA (μM)	Detected by
0% MM	<0.1	<0.1	LC-MS/MS
0% MM	N.D.	N.D.	CE-MS
S. pombe WT CM (10 hours)	3.3	0.6	LC-MS/MS
S. pombe WT CM (20 hours)	17.4	8.5	LC-MS/MS
S. pombe WT CM (30 hours)	55.2	28.4	LC-MS/MS
S. pombe WT CM (30 hours)	68.1	41.5	LC-MS/MS
S. pombe WT CM (30 hours)	59	241	CE-MS
S. pombe WT CM (30 hours)	10	27	CE-MS
*S. pombe fbp1*Δ CM	4.1	14	CE-MS
S. pombe SPACUNK4.10Δ CM	5.3	3.1	LC-MS/MS
S. pombe SPBC1773.17cΔ CM	8.3	3.1	LC-MS/MS
S. pombe SPCC364.07Δ CM	9.1	2.3	LC-MS/MS
S. pombe SPAC186.07cΔ CM	10.6	3.8	LC-MS/MS
*S. pombe*SPAC186.02cΔ CM	7.6	3.1	LC-MS/MS
S. cerevisiae Yea7 CM	98.4	0.9	LC-MS/MS
S. cerevisiae Yea7 CM	129	160	CE-MS
S. cerevisiae Yea8 CM	40.9	0.6	LC-MS/MS
S. cerevisiae Yea8 CM	21	45	CE-MS

Table C. Measured τ, r, a_0, and estimated d

Media	τ ($\pm$ SEM)	$\tau - \tau_0$	r ($\pm$ SEM)	a_0 ($\pm$ SEM)	d ($\pm$ SEM)
0% MM ($n = 15$)	12.93 ($\pm$ 0.25) (τ_0)	-	0.072 ($\pm$ 0.0018)	0.25 ($\pm$ 0.02)	0.34 ($\pm$ 0.01)
WT CM ($n = 22$)	32.77 ($\pm$ 1.03)	19.84	0.056 ($\pm$ 0.0033)	0.23 ($\pm$ 0.01)	0.75 ($\pm$ 0.04)
*fbp1*Δ CM ($n = 16$)	33.37 ($\pm$ 1.31)	20.44	0.057 ($\pm$ 0.0019)	0.28 ($\pm$ 0.02)	0.81 ($\pm$ 0.01)
20 mM HICA ($n = 14$)	14.94 ($\pm$ 0.78)	2.01	0.065 ($\pm$ 0.0017)	0.23 ($\pm$ 0.02)	0.36 ($\pm$ 0.04)
22.5 mM HICA ($n = 20$)	17.90 ($\pm$ 0.70)	4.97	0.064 ($\pm$ 0.0019)	0.23 ($\pm$ 0.02)	0.49 ($\pm$ 0.04)
25 mM HICA ($n = 22$)	29.63 ($\pm$ 1.62)	16.70	0.047 ($\pm$ 0.0020)	0.21 ($\pm$ 0.02)	0.60 ($\pm$ 0.05)
17.5 mM 2K3MVA ($n = 14$)	17.45 ($\pm$ 1.23)	4.52	0.061 ($\pm$ 0.0028)	0.22 ($\pm$ 0.02)	0.43 ($\pm$ 0.04)
20 mM 2K3MVA ($n = 23$)	22.98 ($\pm$ 1.19)	10.06	0.048 ($\pm$ 0.0020)	0.25 ($\pm$ 0.03)	0.45 ($\pm$ 0.03)
22.5 mM 2K3MVA ($n = 17$)	36.55 ($\pm$ 3.16)	23.63	0.031 ($\pm$ 0.0018)	0.24 ($\pm$ 0.02)	0.42 ($\pm$ 0.06)

Table D. Gene ontology terms of biological processes for the differentially expressed 464 genes (p-value < 0.01) between adapted and unadapted cells

GO ID	Term	Corrected p-value	Number of Annotations
GO:0055085	transmembrane transport	2.49E-10	73 (of 353)
GO:0044281	small molecule metabolic process	3.47E-10	108 (of 639)
GO:1901566	organonitrogen compound biosynthetic process	7.36E-09	156 (of 1115)
GO:0002181	cytoplasmic translation	3.52E-08	85 (of 486)
GO:0042254	ribosome biogenesis	8.13E-08	71 (of 379)
GO:0006082	organic acid metabolic process	1.36E-07	64 (of 328)
GO:0019752	carboxylic acid metabolic process	2.18E-07	62 (of 316)
GO:0034220	ion transmembrane transport	2.33E-07	48 (of 213)
GO:0043436	oxoacid metabolic process	2.96E-07	63 (of 326)
GO:0022613	ribonucleoprotein complex biogenesis	5.48E-07	80 (of 469)
GO:0009117	nucleotide metabolic process	3.73E-06	44 (of 201)
GO:1901605	alpha-amino acid metabolic process	5.36E-06	35 (of 141)
GO:1901607	alpha-amino acid biosynthetic process	5.48E-06	28 (of 97)
GO:0006855	drug transmembrane transport	8.22E-06	20 (of 54)
GO:0015893	drug transport	8.22E-06	20 (of 54)
GO:0006753	nucleoside phosphate metabolic process	8.37E-06	44 (of 206)
GO:0009260	ribonucleotide biosynthetic process	8.84E-06	26 (of 87)
GO:0008652	cellular amino acid biosynthetic process	1.12E-05	29 (of 106)
GO:0006811	ion transport	1.36E-05	50 (of 254)
GO:0019693	ribose phosphate metabolic process	1.49E-05	36 (of 153)
GO:0072521	purine-containing compound metabolic process	1.49E-05	36 (of 153)
GO:0055086	nucleobase-containing small molecule metabolic process	1.70E-05	49 (of 248)
GO:0046390	ribose phosphate biosynthetic process	1.95E-05	26 (of 90)
GO:0043603	cellular amide metabolic process	2.14E-05	109 (of 770)
GO:0043604	amide biosynthetic process	2.17E-05	103 (of 714)
GO:0042493	response to drug	4.15E-05	21 (of 64)
GO:0016053	organic acid biosynthetic process	5.17E-05	37 (of 167)
GO:0046394	carboxylic acid biosynthetic process	5.17E-05	37 (of 167)
GO:0009259	ribonucleotide metabolic process	7.14E-05	33 (of 141)
GO:0009152	purine ribonucleotide biosynthetic process	8.39E-05	22 (of 72)
GO:0006164	purine nucleotide biosynthetic process	8.81E-05	23 (of 78)
GO:0072522	purine-containing compound biosynthetic process	8.93E-05	24 (of 84)
GO:0006091	generation of precursor metabolites and energy	0.000109229	30 (of 123)
GO:0017144	drug metabolic process	0.000149196	41 (of 203)
GO:0098656	anion transmembrane transport	0.0001663	26 (of 99)
GO:0042221	response to chemical	0.000221397	57 (of 333)
GO:0042255	ribosome assembly	0.000221525	20 (of 64)
GO:0042273	ribosomal large subunit biogenesis	0.000236141	24 (of 88)
GO:0098739	import across plasma membrane	0.000245616	17 (of 48)
GO:0006525	arginine metabolic process	0.000279633	10 (of 17)
GO:0009199	ribonucleoside triphosphate metabolic process	0.000316376	22 (of 77)
GO:0009150	purine ribonucleotide metabolic process	0.000327206	29 (of 122)
GO:0006163	purine nucleotide metabolic process	0.000406097	30 (of 130)
GO:0006812	cation transport	0.000475913	33 (of 152)
GO:0006086	acetyl-CoA biosynthetic process from pyruvate	0.000487911	6 (of 6)
GO:0009064	glutamine family amino acid metabolic process	0.000531505	16 (of 45)
GO:0098655	cation transmembrane transport	0.00068553	31 (of 140)
GO:0009201	ribonucleoside triphosphate biosynthetic process	0.000751428	16 (of 46)
GO:1901564	organonitrogen compound metabolic process	0.00086918	216 (of 1944)
GO:0009165	nucleotide biosynthetic process	0.000954203	28 (of 121)
GO:1901293	nucleoside phosphate biosynthetic process	0.000954203	28 (of 121)
GO:0044283	small molecule biosynthetic process	0.001257835	52 (of 308)
GO:0009141	nucleoside triphosphate metabolic process	0.001643695	22 (of 84)
GO:0072350	tricarboxylic acid metabolic process	0.002093912	10 (of 20)
GO:0019637	organophosphate metabolic process	0.002427615	55 (of 340)
GO:0009126	purine nucleoside monophosphate metabolic process	0.002530497	22 (of 86)
GO:0009167	purine ribonucleoside monophosphate metabolic process	0.002530497	22 (of 86)
GO:0009142	nucleoside triphosphate biosynthetic process	0.002697376	16 (of 50)
GO:0006099	tricarboxylic acid cycle	0.003845649	9 (of 17)
GO:0046034	ATP metabolic process	0.003884555	19 (of 69)
GO:0043648	dicarboxylic acid metabolic process	0.003962339	18 (of 63)
GO:0009161	ribonucleoside monophosphate metabolic process	0.004359321	24 (of 102)
GO:0006520	cellular amino acid metabolic process	0.006513979	36 (of 193)
GO:0009205	purine ribonucleoside triphosphate metabolic process	0.007745397	19 (of 72)
GO:0009127	purine nucleoside monophosphate biosynthetic process	0.00835637	16 (of 54)
GO:0009168	purine ribonucleoside monophosphate biosynthetic process	0.00835637	16 (of 54)
GO:0006518	peptide metabolic process	0.008362471	91 (of 686)

Table E. List of upregulated genes in the adapted cells: 3% MM with 30mM HICA or 3% MM with 25mM 2K3MVA vs. 3% MM, sorted by HICA+ log2 fold change.
(Abbreviations:L2FC, log2 Fold Change; SE, standard error; p adj, adjusted p-value)

			HICA		2K3MVA	
	ID	Annotation	L2FC (± SE)	p adj	L2FC (± SE)	p adj
1	SPAC977.15	dienelactone hydrolase family, implicated in cellular detoxification	9.71 (± 0.45)	3.1E-99	10.26 (± 0.45)	3.5E-111
2	SPAC750.02c	transmembrane transporter (predicted)	9.38 (± 1.33)	1.2E-10	10.82 (± 1.33)	3.1E-14
3	SPBC1348.05	transmembrane transporter (predicted)	8.35 (± 0.74)	1.5E-27	9.00 (± 0.73)	3.6E-32
4	SPAC977.04	truncated C terminal region of membrane transporter	6.51 (± 1.73)	2.3E-03	7.16 (± 1.73)	3.9E-04
5	SPCC1739.08c	short chain dehydrogenase (predicted)	5.11 (± 0.37)	5.8E-41	6.60 (± 0.37)	5.8E-69
6	SPBC1289.14	adducin (predicted)	5.10 (± 1.54)	9.9E-03	8.25 (± 1.50)	9.6E-07
7	SPBC359.06	*mug14*; adducin, involved in actin cytoskeleton organization	5.06 (± 0.34)	7.1E-48	7.43 (± 0.34)	1.4E-104
8	SPAC11D3.19	Schizosaccharomyces pombe specific protein	4.31 (± 0.93)	8.1E-05	4.66 (± 0.92)	8.5E-06
9	SPAC27D7.03c	*mei2*; RNA-binding protein involved in meiosis Mei2	4.23 (± 0.52)	5.3E-14	4.81 (± 0.52)	3.5E-18
10	SPCC794.01c	*gcd1*; glucose dehydrogenase Gcd1	3.92 (± 0.27)	2.5E-44	5.25 (± 0.27)	5.0E-81
11	SPAC57A7.05	transmembrane transporter (predicted)	3.80 (± 0.25)	1.9E-48	4.11 (± 0.25)	3.1E-57
12	SPBC1683.08	*ght4*; plasma membrane hexose:proton symporter, unknown specificity Ght4	3.75 (± 0.26)	1.5E-43	4.33 (± 0.26)	1.1E-58
13	SPAC977.16c	*dak2*; dihydroxyacetone kinase Dak2	3.67 (± 0.49)	6.2E-12	5.52 (± 0.49)	1.4E-27
14	SPAPB1A11.01	*mfc1*; prospore membrane copper transmembrane transporter Mfc1	3.60 (± 0.36)	6.0E-22	4.78 (± 0.35)	6.5E-40
15	SPCC548.07c	*ght1*; plasma membrane high-affinity glucose:proton symporter Ght1	3.42 (± 0.31)	1.4E-25	4.11 (± 0.31)	2.3E-37
16	SPAC26H5.09c	oxidoreductase involved in NADPH regeneration (predicted)	3.03 (± 0.15)	4.0E-92	3.20 (± 0.15)	2.5E-103
17	SPBC36.01c	spermidine family transmembrane transporter (predicted)	3.03 (± 0.28)	1.2E-25	2.32 (± 0.28)	6.1E-15
18	SPCC1840.12	*opt3*; OPT oligopeptide transmembrane transporter family protein Opt3	2.82 (± 0.18)	2.4E-51	5.08 (± 0.18)	5.0E-177
19	SPAC11E3.06	*map1*; DNA-binding transcription factor, MADS-box Map1	2.69 (± 0.32)	8.3E-15	2.98 (± 0.32)	1.2E-18
20	SPCC584.16c	Schizosaccharomyces specific protein	2.46 (± 0.21)	3.8E-29	2.70 (± 0.21)	1.5E-35
21	SPAC2F7.06c	*pol4*; DNA polymerase X family	2.42 (± 0.62)	1.5E-03	3.13 (± 0.61)	5.6E-06
22	SPAPJ691.02	yippee-like protein	2.33 (± 0.62)	2.5E-03	2.46 (± 0.62)	7.1E-04
23	SPACUNK4.10	*gor1*; glyoxylate reductase (predicted)	2.28 (± 0.19)	1.6E-29	2.84 (± 0.19)	8.8E-46
24	SPBC32C12.02	*ste11*; DNA-binding transcription factor Ste11	2.27 (± 0.23)	3.9E-21	2.79 (± 0.23)	2.7E-32
25	SPBP4H10.09	*rsv1*; DNA-binding transcription factor Rsv1	2.21 (± 0.58)	1.9E-03	2.55 (± 0.57)	1.1E-04
26	SPCC70.04c	Schizosaccharomyces pombe specific protein	2.19 (± 0.55)	1.0E-03	2.98 (± 0.53)	6.0E-07
27	SPBC1683.01	inorganic phosphate transmembrane transporter (predicted)	2.18 (± 0.48)	1.0E-04	2.71 (± 0.48)	3.7E-07
28	SPAC1F8.04c	hydrolase, implicated in cellular detoxification (predicted	2.12 (± 0.40)	2.6E-06	3.63 (± 0.38)	1.3E-19
29	SPBC1289.16c	*cao2*; copper amine oxidase-like protein Cao2	2.11 (± 0.37)	4.2E-07	2.76 (± 0.36)	2.1E-12
30	SPAC31G5.09c	*spk1*; MAP kinase Spk1	2.11 (± 0.25)	2.3E-15	2.43 (± 0.25)	1.1E-20
31	SPCC569.05c	plasma membrane spermidine family transmembrane transporter (predicted)	2.09 (± 0.23)	1.3E-17	2.57 (± 0.23)	9.7E-27
32	SPAC11H11.04	*mam2*; pheromone p-factor receptor Mam2	2.04 (± 0.56)	3.6E-03	2.85 (± 0.56)	7.4E-06
33	SPBC19C7.04c	DUF2406 family conserved fungal protein	2.00 (± 0.28)	7.3E-11	2.12 (± 0.28)	2.1E-12

Table F. Strains used in this study

Organism	Strain name	Genotype	Description	Reference
S. pombe	L972	h$^-$	Wile type (WT)	(9)
S. pombe	AR110	h$^-$ deletion of Chr2:197561-200480	*fbp1*Δ	this study
S. pombe	HN98	h$^-$ SPBC1348.11<<KanMX6-Padh1-mCherry	mCherry-tagged	this study
S. pombe	HN101	h$^-$ SPBC1348.11<<KanMX6-Padh1-mNeonGreen	mNeonGreen-tagged	this study
S. pombe	AR141	h$^-$ SPAC186.07c::*ura4 ura4-D18*		this study
S. pombe	AR142	h$^-$ SPAC186.02c::*ura4 ura4-D18*		this study
S. pombe	AR143	h$^-$ SPACUNK4.10::*ura4 ura4-D18*		this study
S. pombe	AR144	h$^-$ SPBC1773.17c::*ura4 ura4-D18*		this study
S. pombe	AR145	h$^-$ SPCC364.07::*ura4 ura4-D18*		this study
S. pombe	AR168	h$^-$ SPAC977.15::KanMX		this study
S. pombe	AR170	h$^-$ SPAC750.02c::KanMX		this study
S. pombe	AR172	h$^-$ SPBC1348.05::KanMX		this study
S. pombe	AR174	h$^-$ SPAC977.04::KanMX		this study
S. pombe	AR176	h$^-$ SPCC1739.08c::KanMX		this study
S. pombe	AR178	h$^-$ SPBC1289.14::*ura4 ura4-D18*		this study
S. pombe	AR180	h$^-$ *mug14*::*ura4 ura4-D18*		this study
S. pombe	AR182	h$^-$ SPAC11D3.19::*ura4 ura4-D18*		this study
S. pombe	AR184	h$^-$ *mei2*::*ura4 ura4-D18*		this study
S. pombe	AR186	h$^-$ *gcd1*::*ura4 ura4-D18*		this study
S. pombe	AR188	h$^-$ SPAC57A7.05::*ura4 ura4-D18*		this study
S. pombe	AR190	h$^-$ *ght4*::*ura4 ura4-D18*		this study
S. pombe	AR191	h$^-$ *dak2*::KanMX		this study
S. pombe	AR193	h$^-$ *mfc1*::KanMX		this study
S. pombe	AR197	h$^-$ *ght1*::*ura4 ura4-D18*		this study
S. pombe	AR198	h$^-$ *map1*:*ura4 ura4-D18*		this study
S. pombe	AR195	h$^-$ SPCC584.16c::KanMX		this study
S. pombe	FY7755	h$^-$ *cdc25-22*		(10)
S. cerevisiae	OC-2	Wild type	Wine yeast IAM4274, JCM1419	(11)
S. cerevisiae	YEA8	Wild type	Baker's yeast, Lab stock	this study

Table G. PCR primers for RT-PCR used in this study
(Abbreviations: FW, forward primer; RV, reverse primer)

Target genes	FW/RV	Sequence (5'-3')
SPCC1739.08c	FW	CAGCATCGCTACTGCCTTTG
SPCC1739.08c	RV	TCGACGGTATCAGAGCGAGA
SPBC1289.14	FW	TGCGGCATATCGTCTTTTTG
SPBC1289.14	RV	CATCACCCGTAACACCAGGA
SPBC359.06	FW	CTGCAGCTTTTCGCATGTTT
SPBC359.06	RV	TGCTTCCACCAATGATTTCG
SPAC57A7.05	FW	CCATTGGCCGCTAAGACTTC
SPAC57A7.05	RV	ACGGCAATCTGCTCTCCAAT
mfc1	FW	CTTCGGGATTAAGCGGTCTG
mfc1	RV	ACGGCACGAACCAAAAGACT
map1	FW	TTGACCGGGAGCGAAGTAAT
map1	RV	GCTTGGGACGCTTGAGATTC
ght4	RV	AGGTAACGCGGTGATTCAGG
gld1	FW	GCCTCCTCTGATGCCGCTAC

References

1. Chen, S., Zhou, Y., Chen, Y., & Gu, J. fastp: an ultra-fast all-in-one FASTQ preprocessor. *Bioinformatics* **34**, 17, i884-i890 (2018)
2. Wood, V. et al. The genome sequence of *Schizosaccharomyces pombe*. *Nature* **415**, 6874, 871-80(2002)
3. Lock, A. et al. PomBase 2018: user-driven reimplementation of the fission yeast database provides rapid and intuitive access to diverse, interconnected information. *Nucleic Acids Res* **47**, D1, D821-D827 (2018)
4. Li, H., & Durbin, R. Fast and accurate short read alignment with Burrows-Wheeler transform. *Bioinformatics* **25**, 1754-60 (2009)
5. McKenna, A. et al. The Genome Analysis Toolkit: a MapReduce framework for analyzing next-generation DNA sequencing data. *Genome research* **20**, 9, 1297-1303 (2010)
6. Poplin, R. et al. Scaling accurate genetic variant discovery to tens of thousands of samples. *BioRxiv* 201178 (2017)
7. Danecek, P. et al. The variant call format and VCFtools. *Bioinformatics* **27**, 15, 2156-2158 (2011)
8. Patel, V. K. Genotyping by sequencing from sparse sequenced genomes representations from bi-and multi-parental mapping population using a HMM approach (Doctoral dissertation, Universität zu Köln, 2016)
9. Leupold, U. Die Vererbung von Homothallie und Heterothallie bei *Schizosaccharomyces Pombe*. *Comptes rendus des Travaux du Laboratoire Carlsberg* **24**, 381-480 (1950)
10. Yoshida, S., Al-Amodi, H., Nakamura, T., McInerny, C. J., Shimoda C. The Schizosaccharomyces pombe cdt2(+) gene, a target of G1-S phase-specific transcription factor complex DSC1, is required for mitotic and premeiotic DNA replication. *Genetics*, **164**, 3, 881-93 (2003)
11. Takeda, M., Nakazato, A., Goseki, M., & Tsukahara, T. Classification of *Saccharomyces cerevisiae* OC no. 2 of wine yeast. *Commemoration number of the 90th Anniversary of Foundation.* 125-128 (1981)
