# Supplementary material for: Autotoxin-mediated latecomer killing in yeast communities
Source: PLoS Biol. 2022 Nov 7;20(11):e3001844. doi: 10.1371/journal.pbio.3001844 (PMC9639812; doi:10.1371/journal.pbio.3001844)
Supplement: Attachment — Submitted filename: 220804_response.pdf [file pbio.3001844.s035.pdf]

Overall, we rewrote the manuscript as a “short report” to focus on the report of the new phenomenon. We added some new results including the measurements of HICA and 2K3MVA in the conditioned media (see Supplemental Table 2). Their concentrations in the conditioned media were much lower (55-68 μM at 30 h) than that we have added as a single dose. This means there may be additional factors in the CM to enhance their effects. Indeed, the single dose of 50 μM HICA or 2K3MVA to the CM caused the growth delay (Figs. 2H and I). Further, the delay phase was reduced in CMs from mutants producing less HICA and 2K3MVA (Supplemental Fig. S12), and adding back 25-50 μM of HICA or 2K3MVA (equivalent to their concentrations in wild-type CMs) to these CMs rescued the delay phase (Supplemental Fig, S13). Those results suggest that HICA and 2K3MVA are indeed the important entities of the autotoxins that can work in a single dose, while they need other components for their full actions. Since treatments of the CM with autoclaving, DNase, RNase, and proteases did not affect its ability to cause the growth delay (Supplemental Fig 9), such additional components should not be volatile/thermolabile materials, DNA, RNA, and proteins. Accordingly, we have therefore decided to focus the present paper on reporting new phenomena and revise our conclusion that HICA and 2K3MVA are important elements of the autotoxin, but that this phenomenon is not simple due to HICA and 2K3MVA.

Below we address the corrections suggested by the reviewer in a point-by-point manner. Note that the reviewer’s comments are noted with brackets, and the corrections are highlighted by red in the revised manuscript.

Reviewer #1:

[In this study titled 'Autotoxin-mediated voluntary triage in starved yeast community', the authors identify a novel phenomenon wherein S. pombe cell populations adapt to glucose starvation by releasing autotoxins. The authors identify two compounds (HICA and 2K3MVA) that putatively act as autotoxins driving this collective cellular phenomenon. Only a fraction of the cells in the population survive as these autotoxins build up. Interestingly, this phenomenon seems to be evolutionarily conserved, even in the distantly related S. cerevisiae cells, suggesting some universality.

This study reports a novel, interesting phenomenon. However, this study definitely overstates many claims, or makes plausible inferences without sufficient data.

Upfront, the highlight of the study is the discovery of this collective phenomenon mediated by produced metabolites that are accessible to all cells, but leading to some cells dying and others selectively surviving. Three major points are unclear: (i) whether such a phenomenon provides any advantage to cells within the population (and what it might mean to a population of clonal cells), (ii) while there is no molecular/mechanistic

insight (and let me state that there need not always be molecular/mechanistic insight) into how this phenomenon might be regulated, it is not clear even from the data presented whether the phenomenon is purely a reproducible, stochastic event, or if there are any deterministic features that would determine which cells might be resistant to auto-toxins, or might benefit from these molecules, and (iii) is there a separation within the population into cells that produce the toxins vs others that do not produce toxins/are resistant to toxins/consume
toxins. In may ways, this study works better as a 'short report' that highlights this phenomenon, rather than a full article that does not provide mechanistic or conceptual insight into how such a phenomenon might occur.]

Thank you for your positive evaluation of our manuscript and constructive comments.
Since we focused on reporting the new phenomenon in this paper, we reconstructed the manuscript as a "short report" according to your suggestion.

[The following are the major concerns with this study.

Major concerns
1. The authors suggest that the presence of inhibitory compounds and not nutrient depletion is responsible for the delay in growth observed in cells in CM. They show that glucose addition rescues this delay in growth suggesting that the inhibitory effect is absent when glucose is present (fig 2A). However, this experiment does not rule out the possibility that glucose-depletion itself is responsible for the delay in growth after shift to CM. The fbp deletion experiment does not answer this directly. What happens if an alternate 'good' carbon source (eg. glycerol) or an amino acid mix/glutamate is added to the CM? Will the delay in growth be rescued? This experiment will tell us whether the growth delay is independent of all nutrient availability or not. It will also strengthen the observation that the rescue in growth is glucose specific.]

Thank you for your constructive comments.
First, we would like to mention it is clear that glucose deprivation alone does not cause growth retardation, since different concentrations of HICA and 2K3MVA added to the same glucose-depleted medium cause a dose-dependent delay. According to your suggestions, we added various species of sugars as a carbon source, an amino acid supplemental mixture, or glutamate to the CM and measured the growth of cells. Then, we found that the behavior was depending on what we added: Fructose rescued the delay in growth as much as glucose, and mannose and galactose rescued partially. In addition, we also added 2-deoxy-D-glucose according to Reviewer 4's suggestion and confirmed that cells did not grow. These results suggest that the rescue of the growth depends on the metabolism of sugars, but not on specific signaling related to

glucose. Further, an amino acid mix and glutamate were not able to rescue the delay and had almost no effect on cellular growth. This suggests that amino acids have little effect on autotoxin-induced cell death. We added Supplemental Fig. S4 and an explanation (line 54) for those experiments.

[2. It is not clear from the data at what point in time do cells in 0% MM start producing HICA and 2K3MVA. Are these present only in CM or do cells which are shifted to CM continue to produce these compounds? Can the amount of these compounds be measured over time to address this?]

We measured the concentrations of HICA and 2K3MVA in the CM sampled at times 0, 10, 20, and 30 hours and found that those concentrations increased over time. We described the concentrations of HICA and 2K3MVA in the CM in Table S2 and described this point (line 141). The results suggest that cells continuously produce those toxins.

[*3. Relatedly, is there a build up and then a decrease in these metabolites? If the compounds only accumulate, then the interpretation is different from if the metabolites accumulate and then start decreasing (suggesting some utilization and/or breakdown), which holds even if the metabolites in the medium flat-line. An additional possibility is that there is a feeder-utilizer type phenomenon that is established, where some cells continue to put out these metabolites, while others do not produce it (and possibly also start utilizing these metabolites). Can the authors establish which of these possibilities exist? If so, this becomes conceptually very interesting (from the perspective of collective cell behavior), regardless of which process occurs.*]

Thank you for your suggestions.
We are also interested in how cells produce or decompose the autotoxins. If cells grow, however, synthesis of the autotoxin will increase, not only decomposition. Therefore, we need to develop new methodologies to measure production and decomposition separately. But we could obtain clues for this question from our time course quantification of HICA and 2K3MVA. Their concentrations increased more than five times from 10 to 20 hours, and more than three times from 20 to 30 hours (Supplemental Table S2). Since the cell doubling time in 0% MM was about 10 hours (Supplemental Table S3), the concentrations of these molecules increased faster than cells, indicating that these molecules rapidly accumulated in the media. Therefore, it was speculated that the degradation or consumption of these molecules, even if they were present, would be limited. We added a remark on this point (line 141).

[4. *Relatedly, the authors can definitely identify cells that now are able to grow in medium which as these autoinhibitors. Can these cells actually start utilizing/breaking down these metabolites? A possible experiment would be to separate the cells that are now growing in this medium, and then add conditioned medium (exogenously) to these cells, and see if these metabolites are taken up and consumed. This experiment would be quite important in order to understand what is happening with this autoinhibitory system (and interpretations therin).*]

Thank you for suggesting intriguing experiments. Although they are interesting experiments, we regret to say that a precise analysis of the processes by which individual cells continue to produce or utilize toxins after adaptation would require the introduction of new methodologies and is beyond the scope of this short report.

[5. *(points 5-7 are related queries) Do the authors have any idea how these compounds (eg. HICA) are produced in cells? Eg. Is there any evidence of high branched-chain amino acid catabolism or keto-genesis, or is this just a reflection of an accumulation of BCAAs (which are all ketogenic)? Can, for example, just the addition of leucine/BCAAs after glucose depletion result in this phenomenon of cells dying?*]

How HICA and 2K3MVA are synthesized has not been reported in *S. pombe*. However, as we will describe in more detail later, we have obtained novel mutants defective in the production of those compounds.
We also investigated how the addition of BCAAs affects cell death. We had already observed the effect of adding the mixture of leucine, adenine, uracil, and histidine, because those compounds are commonly used as auxotrophic markers in the genetics of *S. pombe*, and had confirmed that they did not cause cell death (see Supplemental Fig. S2). Further, to observe leucine alone, rather than the mixture, we solely administrated leucine and BCAAs and confirmed that they did not cause cell death.
We have added those results (Supplemental Fig. S11) and explanation (line 127).

[6. *Can the production of these compounds be disrupted genetically? Will these mutant cells behave differently from the WT cells in terms of glucose starvation response?*]

According to your suggestion, we tried to construct mutants for the production of these compounds as mentioned above. Although synthetic pathways producing HICA and 2K3MVA have been unknown in *S. pombe*, a hydroxyacid dehydrogenase was identified as a synthetase for HICA in bacteria (Chambellon *et al.*, *J Bacteriol*, 2009 for *Lactococcus lactis*). Neither its homolog nor any enzymes for HICA and 2K3MVA synthesis have been reported in *S. pombe.* Thus, we searched genes encoding hydroxyacid dehydrogenase, whose targets are unknown in *S. pombe*, by using UniProt, and made mutant strains lacking these genes.

Then, the concentrations of HICA and 2K3MVA in media conditioned by all of these strains decreased but did not reach zero. These results suggest that *S. pombe* synthesized HICA and 2K3MVA by using multiple pathways and synthetases. Further, we confirmed that the delay phase of WT cells cultured in these CM was shortened. On the other hand, the behaviors of those mutants in the glucose-depleted media and their responses to autotoxins were almost similar to those of WT.
We have added those results (Supplemental Fig. S12 and S19) and explanations (lines 155 and 207).

[7. *Relatedly, in complementary experiments, if the authors keep replacing the medium with glucose depleted medium without the HICA and the keto-acids, will cells in the population now start dying/growing in a similar manner? This relates to the idea of whether it is actually HICA that causes cell death, or is it just the accumulation of leucine that is sufficient for this phenomenon. Leucine is a ketogenic acid, and also can be metabolized to HICA. So this question of whether this phenomenon is a consequence of just high accumulation of leucine, or whether this is truly a produced metabolite specific phenomenon becomes interesting and important. It can also help separate possible signaling roles of leucine vs metabolic roles, that cause this phenomenon.*]

First, we would like to mention that *S. pombe* cells adapted to glucose starvation can grow in the glucose-depleted medium in the absence of HICA and the keto-acids as reported in our previous paper (Hirota et al., *Nature* 2008). To conduct your suggested experiments precisely, it would be necessary to examine the single cell behavior after the medium change in microchamber-based chemostat experiments, which is beyond the scope of this short report.
Regarding the effects of leucine, we designed a different experiment that the cell precultured in the glucose-rich medium are shifted to the minimal medium without glucose. In such an experiment, the delay phase was not observed in the presence or absence of leucine/BCAAs (Supplemental Fig. S11). Furthermore, since the delay phase was observed in both of the CM with or without supplements for yeast auxotrophic markers including leucine (Supplemental Fig. S2), it is plausible that BCAAs including leucine in the media do not contribute much to the occurrence of the delay phase.

[8. *The authors show that addition of glucose prevents the growth delay in cells shifted to CM (Figure 2A). Is this because of upregulating the same set of genes that help the cells cultured in HICA and 2K3MVA to reduce the growth delay (Figure 5A)? Can the authors determine if the major genes identified for adaptation in this study (SPCC1739.08c etc) are upregulated once glucose is added to the CM? This will strengthen the story and also address point number 1.*]

According to your suggestion, we confirmed that not all but most of the genes that were upregulated in glucose-rich media with an autotoxin were also upregulated in the CM with glucose by RT-PCR (Supplemental Fig. S22). Thus, a similar adaptation system might work for the growth in the CM with glucose. However, both the autotoxins and conditioned media cause cell death via two processes, apoptosis and necrosis as shown in Supplemental Fig. S17 in the revised manuscript. It suggests that the mechanism of cell death is not simple but multifarious, and there is no uniformity in the way cell dies. Therefore, it is expected that the recovery mechanism also varies depending on different cell fates. We would like to investigate further mechanisms of growth rescue by glucose addition in future studies.
We described those points in the text (lines 190 and 232).

[9. *There is no data to establish unambiguously that the adaptation to autotoxins is epigenetic. Can it be just a transcriptional regulation where the adaptive genes are upregulated? Is this an inheritable phenotype (i.e. the resistant cells which grow remain resistant across generations)? This is the classical definition of epigenetic, and if this is not the case, then much of this can be just attributed to stochasticity in gene expression (and therefore heterogeneity). Is there a specific type of gene expression signature (that can come from stochastic events) that will provide resistance to cells?*]

If cells do not memorize the adapted state, divided cells will not survive after the delay phase, and only a small portion of cells can escape death and grow. Then, the growth rate would decrease eventually. However, after the delay phase, the growth rate in the CM was stably maintained. Hence, those growing cells should inherit the adapted state from those mothers, where such an inherited state would not involve genetic changes. We thought this feature is very similar to the classical definition of epigenetics, as you pointed out. However, such a description might be misleading for some readers, and we decided to delete the term "epigenetics" in the revised manuscript. Inherited adapted states of cells might change stochastically. However, to investigate this, a single-cell measurement should be employed to monitor the physiological states of each cell, which is rather beyond the scope of this study. We have carefully added a detailed explanation on this point (line 65) and rewrote the text.

[10. *The authors suggest that a few cells in the population adapt to the autotoxins epigenetically, possibly by upregulating genes for detoxification and toxin export. However, it is not clear whether these genes are specifically upregulated only in a few cells in the population. The data presented shows that the cells that are pre-exposed to toxins have these genes upregulated. However, it is possible that all the cells in CM have high expression of these genes, or there is stochasticity in gene expression (see point 7). Can the authors do conclusive experiments to determine if these genes are specifically upregulated in the few*

cells that have better survival in CM? One possibility is to use a fluorescent reporter under promoters of the major upregulated genes, and correlate the expression of the reporters with the cells that live/die. This would more unambiguously establish the phenomenon and conceptual mechanism that enables the phenomenon.]

The variation between survived and dead cells in the presence of autotoxins is indeed an interesting question. For the extensive study, a single-cell measurement or single-cell RNA-seq (such as the 10x Genomics system) would be informative. However, we again have to mention that these experiments are beyond the scope of the current study, and we would like to try them in the future study.
Instead, we investigated the cell-to-cell heterogeneity depending on the cell cycle. We measured the death rate of the strain with a temperature-sensitive *cdc25-22* mutation, which can arrest the cell cycle in the G2 phase at a higher temperature. Using *cdc25-22* mutants, we prepared synchronously dividing cells and found that they showed higher death rates than asynchronously dividing cells, which will contain a substantial portion of cells arrested in the G0 phase. This suggests that the cell cycle stage is one of the important factors in determining the cell competency to survive or die, and the resting cells may show tolerance to autotoxins. We have added Supplemental Fig. S18 and a remark explaining this point to the text (line 199).

[*11. "Thus, the toxin producer is lost to a cheater, which only has the antitoxin system, whereas the cheater loses to cells having neither any toxins nor immunity [27]. In contrast, the voluntary triage does not cost much because the adaptation mechanism is usually offed without the toxin. This suggests that voluntary triage is resistant to cheaters" lines 187-191*
In theory, cheaters which have upregulated genes for export and detoxification (the genes identified in this study) can survive the autotoxins without necessarily producing the autotoxins themselves. Can the authors clarify this? This goes back to the earlier question of whether the population actually splits into toxin producers vs non-producers/consumers, vs all cells producing (and sensing) the putative auto-toxins.]

As you pointed out, theoretically, cheaters would survive if they have a protection mechanism against toxins. Cheaters can easily survive under glucose-depleted conditions if they have a protection mechanism against toxins. However, if cheaters invade, since they do not need to pay the cost for the production of toxins, the cheater will dominate the population under nutritional-poor conditions. In this case, the toxin-producing cells cannot survive unless they continue to evolve to produce different toxins. However, under nutritional-rich conditions, cheaters have disadvantages in their stable expression of protection mechanisms. When the environment fluctuates between glucose-rich and glucose-depleted conditions like real natural conditions, cheaters would be expelled eventually. Indeed, in the case of the antitoxin system, an “arms race” between toxin and resistance has been observed, but the observed autotoxins are conserved among distant species,

suggesting that there is no arms race. In addition, in the population dynamics theory and simulation, we found that cells with the autotoxins and adaptation mechanism can survive even in the presence of cheaters. We would like to pursue more detailed theoretical aspects in future studies. We have added a more detailed explanation of this point and toned down the discussion (lines 269, 280, and 293).

[Minor points

1. *The figures, especially the graphs can be labelled in more detail to help the readers understand the figures. The figures 3 B, C, D, E could be labelled in a better way. Since there are multiple plots in this paper, proper labelling will help the readers understand the figures and graphs better.*]

We modified the labels in Fig. 2B-E as you pointed out.

[2. *The title seems incomplete. There is some kind of autotoxin mediated triage….but? Triage leads to what? Triage exists? Is it really voluntary? I hope the authors give this some more thought.*]

We changed the title to “Autotoxin-mediated latecomer killing in yeast community during glucose depletion”.

Below we address the corrections suggested by the reviewer in a point-by-point manner. Note that the reviewer's comments are noted with brackets, and the corrections are highlighted by red in the revised manuscript.

Reviewer #2:

[*In this article, the authors describe a phenomenon where fission yeast release toxins (identified here using mass spectrometry as primarily leucic acid--2-hydrozyisocaproic acid, HICA and L-2keto-3methyvalerate--2K3MVA) under glucose starvation conditions. The discovery of HICA is interesting, as it has been shown to have antifungal and antimicrobial populations in other microbial studies, including of Lactobacillus. The authors term this toxin release voluntary triage. Cells that adapt to conditioned media (CM) containing the toxins can outcompete unadapted isogenic cells. The authors perform RNA-seq and find evidence that adaptation is due to expression of genes involved in transport (in addition the authors verified with genomic DNA-sequencing that survival in conditioned media was not due to accumulation of mutations). In addition, comparison with two S. cerevisiae strains indicates that these autotoxins and voluntary triage may be a conserved mechanism between distant yeast species.*

This is an interesting paper that may have implications for understanding microbial community dynamics, population dynamics, and multicellularity. However, I feel that the paper is underdeveloped and I do not have a good sense for the importance of the autotoxins in dictating population-level behavior (including heterogeneity in cell death) nor whether I should think about toxin production as a regulated process or a chance byproduct of metabolism under glucose-deprived conditions. I have some suggestions that should be addressed prior to publication:]

Thank you for your positive evaluation of our study and constructive comments.

[Major Comments:

* *The paper is written in a way that seems to imply the release of HICA and 2K3MVA are regulated processes or cellular "decisions", but is this an appropriate way to think about this process? I'm guessing that these molecules are being generated due to catabolism occurring in conjunction with starvation, so should I be thinking of this as anything more sophisticated than yeast having to take less desirable metabolic routes as the environment worsens, leading to the generation of toxic byproducts?*]

As you mentioned, our previous manuscript would give a misleading impression to readers as if cells were performing triage with their decisions, and it was anthropomorphizing cells too much. As you guessed, cells

may have initially released autotoxins as a result of undesirable catabolism. However, please note that it is compatible with our experimental results, in which those autotoxins worked to kill the latecomer in competition. Indeed, even in distant species, cells have evolved to adapt to the autotoxins rather than not produce them.

Thus, following the suggestion of Reviewer 3, we have changed the name "voluntary triage" to "latecomer killing," which is more appropriate to the phenomenon. Besides, we changed the title to “Autotoxin-mediated latecomer killing in yeast community during glucose depletion” and reconstruct the manuscript as a “short report” following the editor’s and Reviewer 1’s suggestion to focus on reporting the new phenomenon.

[* *What is the mechanism of toxicity by the identified autotoxins? Why are the toxins only toxic in the absence of glucose?*]

We analyzed how cells are killed by the autotoxins or CM by co-staining of annexin-V (AnnV) and propidium iodide (PI): Early apoptotic cells exhibiting phosphatidylserine externalization and are stained by AnnV but not by PI. In contrast, primary necrotic cells show ruptured plasma membranes which are stained by PI but not by AnnV. Late apoptotic and secondary necrotic cells are stained by both AnnV and PI.

Both of the autotoxins and conditioned media cause cell death via two processes, apoptosis and necrosis as shown in Supplemental Fig. S17 in the revised manuscript. It suggests that the mechanism of cell death is not simple but multifarious, and there is no uniformity in the way how cell dies. We have added the figure (Supplemental Fig. S17) and explanation (line 190).

We agree that it is an interesting question why glucose addition restores cell survival. Since some genes required for the adaptation to autotoxins were also upregulated in the CM with glucose, they might be one of the mechanisms to rescue cell death in the presence of glucose (Supplemental Fig. S22). However, as we described above, the cell death mechanism is multilayered, and it is expected that the recovery mechanism also varies depending on different cell fates. We would like to further investigate mechanisms for cell growth rescue by glucose addition in future studies.

[* *Given that there seems to be the suggestion that toxin production and adaptation prevents mass-suicide (see Intro, Discussion) it would seem important to understand the nature of heterogeneity of entry into the stress-resistant states. The introductory sentences (line 17-22) would seem to suggest that as glucose is depleted cells differentiate into adapted and non-adapted cells, and the non-adapted cells will go on to die. Is there evidence for this? For example, how coherent is expression of the identified genes as cells progress into glucose starvation?*]

The variation between survived and dead cells in the presence of autotoxins is indeed an interesting question. For the extensive study, a single-cell measurement or single-cell RNA-seq (such as the 10x Genomics system) would be informative. But these experiments are beyond the scope of the current study, and we would like to try them in future studies. Instead, we investigated the cell-to-cell heterogeneity depending on the cell cycle. We measured the death rate of the strain with a temperature-sensitive *cdc25-22* mutation, which can arrest the cell cycle in the G2 phase at a higher temperature. Using *cdc25-22* mutants, we prepared synchronously dividing cells and found that they showed higher death rates than asynchronously dividing cells, which will contain a substantial portion of cells arrested in the G0 phase. This suggests that the cell cycle stage is one of the important factors in determining the cell competency to survive or die, and the resting cells may show tolerance to autotoxins. We have added Supplemental Fig. S18 and a remark explaining this point to the text (line 199).

[* *I find the choice of the term "voluntary triage" confusing. It seems to imply that cells are choosing to make toxic molecules. Or that this is an evolved mechanism.*]

As we described above, we have changed the term "voluntary triage" to "latecomer killing".

[* *How should I understand the similarity between Figure 1B and 1C? Since the fbp1 mutant cannot grow further in glycerol, when you switch to 0% MM are you adding more cells for fbp1 than for WT? Or are the fbp1 cells somehow producing the same amount of toxin over the many hours (up to 30 h) of incubation in 0% MM to produce CM despite presumably growing less in the 0% MM media?*]

We are also interested in why the medium conditioned by the *fbp1* mutant caused a similar delay phase. Concentrations of HICA and 2K3MVA in the CM of the *fbp1* mutant were lower than those in the WT CM (Supplemental Table S2). From these results, we hypothesized that the *fbp1*Δ CM contains substantial amounts of cofactors for autotoxins that are not effective alone but can enhance the action of HICA and 2K3MVA. Consistent with this notion, the observed concentrations of HICA and 2K3MVA in the wild-type CM were much lower (55-68 μM) than those we used for the addition of them with a single dose.
This means there may be additional factors in the CM to enhance their effects. Indeed, the single dose of 50μM HICA or 2K3MVA to the CM caused the growth delay (Figs. 2H and I). Further, the delay phase was reduced in CMs from mutants producing less HICA and 2K3MVA (Supplemental Fig. S12), and adding back 25-50 μM of HICA or 2K3MVA (equivalent to their concentrations in wild-type CMs) to these CMs rescued the delay phase (Supplemental Fig, S13). Those results suggest that HICA and 2K3MVA are indeed the important entities of the autotoxins that can work in a single dose, while they need other components for

their full actions. We would like to identify such additional factors in future work. We added a detailed explanation of these points (line 149).

[*   *Can you clarify how the search for the inhibitors was done? The methods say that the 12 were added at 10-40mM. Why was this the relevant concentration range? Particularly, given that later in the paper it looks like HIC and 2K3MVA were added at substantially lower concentrations.*]

As described above, we tried to identify major candidate substances that are effective with a single dose as the first step in understanding the new phenomenon. To certainly identify the candidates, we started to test sufficiently high concentrations range (10-40 mM) of the compounds. We have added the explanation on this point (line 385).

[*   *Its not clear to me that the statements made in line 180-183 are supported by the evidence. What counts as a mass suicide? Is there evidence that more cells would die without the production of the autotoxins? Similarly, the following comments about relative susceptibility to invasion by cheaters (lines 187-190) do not seem supported nor tested in the current work.*]

There is no evidence that the production of the autotoxins would increase the survived population under the glucose-depleted condition. Therefore, we toned down that part (line 265).
However, we consider that combination of the autotoxins and adaptation will be relatively susceptible to invasion by cheaters (lines 272, 284, and 297). Cheaters can easily survive under glucose-depeleted conditions if they have a protection mechanism against toxins. However, if cheaters invade, since they do not need to pay the cost for the production of toxins, the cheater will dominate the population under nutritional-poor conditions. In this case, the toxin-producing cells cannot survive unless they continue to evolve to produce different toxins. But under nutritional-rich conditions, cheaters have disadvantages in their stable expression of protection mechanisms. When the environment fluctuates between glucose-rich and glucose-depleted conditions like real natural conditions, cheaters would be expelled eventually. Indeed, in the case of the antitoxin system, an “arms race” between toxin and resistance has been observed, but the observed autotoxins are conserved among distant species, suggesting that there is no arms race. In addition, in the population dynamics theory and simulation, we found that cells with the autotoxins and adaptation mechanism can survive even in the presence of cheaters. We would like to pursue more detailed theoretical aspects in future studies. We have added a more detailed explanation of this point and toned down the discussion (lines 269, 280, and 293).

[*Minor Comments:*

*	*Line 15-It is not clear in this context what it means for cells to "tag" themselves. (Also line 67).*]

Although we described changes in the cell state to an adaptive one as “tag”, this may not be clear to some readers. Therefore, we changed this word (line 15, 65).

[*	*The shorthand for the media (3% MM is MM with both 3% glucose and 3% glycerol) is confusing.*]

Although we added 3% glycerol to all the media, the lack of description may be confusing, as you pointed out. Therefore, we emphasized the addition of 3% glycerol to all the media (line 28) and Figure 1A.

[*	*Supplemental Figure 2: Does the delay phase really look the same? It seems longer without amino acids.*]

We measured the growth curves for more biological replicates and confirmed that the addition of amino acids had little effect on the delay phase.

[*	*Figure 2: It is very hard to see some of the lines, especially the light blue line for fbp1CM+GLC (in both panel B and D)*]

We changed the color combination and plotted thicker lines to improve the visibility (Fig1 E and G).

[*	*Line 112: Is the point of this line that the effective concentration of HIC and 2K3MVA required to inhibit cell growth is higher than one would expect given the mass spec data? The meaning of this sentence is not totally clear to me. And when the molecules are combined, how do the necessary inhibitory concentrations compare to the "natural condition"?*]

As mentioned at the beginning of the response letter, there was a difference between the observed concentrations of HICA and 2K3MVA in the CMs and the concentrations of added these compounds. We have already explained the possible reasons for this and revised the corresponding sentences (line 130).

[* *Line 190: What does it mean "the adaptation mechanism is usually offed without the toxin?"*]

Thank you for letting us know about this typological error. We changed that sentence to “the adaptation system can be turned off without the toxin” and added a detailed explanation on this point (line 268). When cells precultured without autotoxins (e.g., glucose-rich media without HICA and 2K3MVA) were transferred into glucose-depleted media with the autotoxins (e.g., CM or glucose-depleted MM with HICA or 2K3MVA), cells died. It indicated that the adaptation mechanism does not work in the absence of HICA or 2K3MVA.

[* *Line 228-234—these methods are not clear*]

We refined the methods section (line 319).

[* *Line 254—I think that the strain info is actually in Supplementary Table 5*]

We corrected the reference for the Table (line 335).

[* *Line 266—missing Figure reference*]

We added the reference of the Figure (line 369).

[* *Why do so many of the growth curves show periodic fluctuations? (e.g. red line Supp Fig 1)*]

A source of such periodicity is considered to be the periodic fluctuation of AC electricity since we also detected similar periodic fluctuations of OD even when we measured water without cells. The figure attached below shows OD of water measured by three different sensors measured at the same time, which was converted from the voltage on sensors into OD600 value. When OD is sufficiently high, such a basal fluctuation is masked. Therefore, this phenomenon is an artifact and does not affect our conclusion.

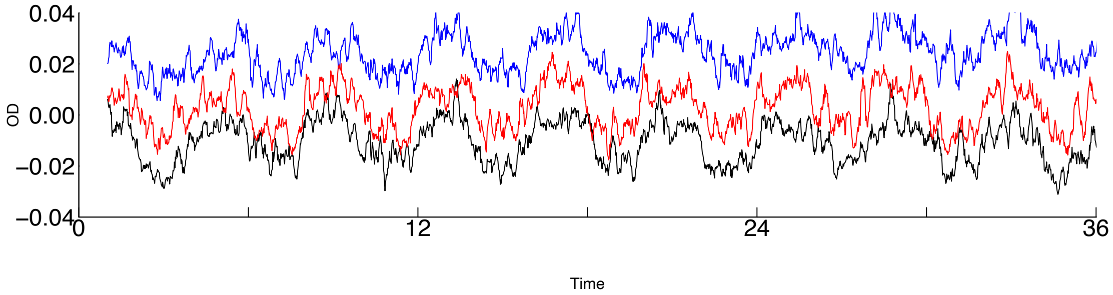

[* *this be? And secretion does not require growth?*]

Although some sentences are missing, we assume that you probably asked whether or not cell growth is necessary for HICA or 2K3MVA secretion. The answer is not necessary, since we detected an accumulation of HICA and 2K3MVA in CMs from the *fbp1*Δ mutant which does not grow in the absence of glucose.

[* *Line 216—Reference 34 does not seem to be the correct reference here.*]

We have cited a correct reference (line 304, reference 37).

Below we address the corrections suggested by the reviewer in a point-by-point manner. Note that the reviewer's comments are noted with brackets, and the corrections are highlighted by red in the revised manuscript.

Reviewer #3:

[*In this manuscript, the authors report an interesting phenomenon of autotoxin-mediated killing in yeasts. They show that switching the carbon source from glucose to glycerol results in the secretion of molecules that can kill yeast cells not previously exposed to the glycerol medium. This novel discovery opens an unexpected avenue into understanding intercellular communications in unicellular organisms.*]

Thank you for your positive evaluation of our study and constructive comments.

[*I have the following suggestions for the authors to consider.*

Major point

I am not fully convinced that HICA and 2K3MVA are the major components of autotoxins. The authors should provide measurements or estimation of the concentrations of HICA and 2K3MVA in the conditioned media as this information is important for interpreting the experiments performed using synthetic compounds.]

As mentioned at the beginning of the response letter, we added some new results including the measurements of HICA and 2K3MVA in the conditioned media (see Supplemental Table 2). Their concentrations in the conditioned media were much lower (55-68 μM at 30 h) than that we have added as a single dose. This means there may be additional factors in the CM to enhance their effects. Indeed, the single dose of 50μM HICA or 2K3MVA to the CM caused the growth delay (Figs. 2H and I). Further, the delay phase was reduced in CMs from mutants producing less HICA and 2K3MVA (Supplemental Fig. S12), and adding back 25-50 μM of HICA or 2K3MVA (equivalent to their concentrations in wild-type CMs) to these CMs rescued the delay phase (Supplemental Fig, S13). Those results suggest that HICA and 2K3MVA are indeed the important entities of the autotoxins that can work in a single dose, while they need other components for their full actions. We added a detailed explanation of this point (lines 130 and 155).

[*In addition, the authors did not rule out secreted proteins in the conditioned media as contributing factors. I suggest the authors test whether denaturing proteins by heating affects the cell-killing activity of conditioned media.*]

According to your suggestion, we autoclaved the conditioned media, and we still observed the delay phase. Furthermore, neither the addition of proteases, DNase nor RNase affected the delay phase. Thus, we believe that we have ruled out the possibility that peptides, proteins or nucleic acids, and volatile/thermolabile materials are entities of the autotoxins. We have added those results (Supplemental Fig. S9) and explained this point (line 99).

[*Minor points*

(1) The term "voluntary triage"
I think the term "voluntary triage" gives an impression that a fraction of cells shifted to the glycerol medium are killed by autotoxins. But this is not be the case. Rather, cells that can be killed by autotoxins are naive "latecomer" cells not previously exposed to the glycerol medium. I suggest the authors consider using a different term, for example, "latecomer killing", to more accurately describe the phenomenon.]

We changed the term “voluntary triage” to “latecomer killing”, following your suggestion.

[(2) *The words "starved", "starvation", and "starving"*
Title
"Autotoxin-mediated voluntary triage in starved yeast community"
Abstract
"we demonstrate an elaborate response of the yeast community against glucose starvation, named the voluntary triage"

"Glucose starvation" usually refers to shifting cells from glucose-containing medium to a medium lacking any carbon source. In this study, the condition used to induce autotoxins is shifting cells from glucose-containing medium to glycerol-containing medium. I think it is not appropriate to call this treatment "glucose starvation".

The authors may want to consider changing "starved yeast community" in the title to "yeast community adapting to a poor carbon source".

For the above sentence in the abstract, I suggest changing it to "we demonstrate an elaborate response of the yeast community to a shift to a poor carbon source and name this response the voluntary triage".

Elsewhere in the manuscript, the words "starved", "starvation", and "starving" also need to be re-considered.]

We have carefully altered the corresponding words to contextually appropriate ones, e.g., “starvation” was changed to “depletion”.

[(3) *Are cells producing autotoxins sensitive to autotoxins?*
Abstract
"yeast cells release autotoxins, such as leucic acid and L-2keto-3methylvalerate, which can even kill the cells producing them"
It is not clear to me how the authors can conclude that autotoxins kill the cells producing them. I think it is likely that cells producing autotoxins are resistant to killing.]

We have changed "yeast cells release autotoxins, such as leucic acid and L-2keto-3methylvalerate, which can even kill the cells producing them" to "yeast cells release autotoxins, such as leucic acid and L-2keto-3methylvalerate, which can even kill the *clonal* cells *of the ones* producing them".

[(4) *How are conditioned media prepared?*
The following description is insufficient.
"Cells were precultured in 3% MM and transferred to 0% MM and cultured. The supernatants were then filtered using a 0.22 µm PVDF membrane (Millex Sterile Filter Unit, Merck Millipore, Burlington, MA, USA)."
The authors should provide more details, including the starting OD600 in 0% MM and the OD600 at the 30 h time point. Because fbp1Δ cells do not grow in 0% MM, I wonder whether the authors might have used different starting OD600 for WT and fbp1Δ.]

We added an explanation to the Materials and method section (line 351). The starting OD600 is approximately 0.1-0.2. and the final OD600 differs between strains.

[(5) *The sources of synthetic autotoxin compounds*
The authors should provide information on the sources from which enantiomers of HICA and 2K3MVA were

obtained or how they were synthesized.]

We purchased purified enantiomers of HICA and 2K3MVA for the experiments. We added the information of the reagents for these compounds and other candidate compounds to the subsection of “Search for growth inhibitors” in the Materials and methods section (line 387).

[*(6) Are genes important for adaptation to HICA also important for acquiring resistance in 0% MM?*
It is useful to test whether genes such as SPAC57A7.05Δ are important for acquiring the ability to resist to conditioned media through preculturing in 0% MM.]

We added that experiment according to your suggestion. Seven deletion mutants (described in Fig3L) were precultured in 0% MM, and their growth was measured in WT CM. All of their growth was almost the same speed as that of WT. This result suggests that these genes were important for adaptation to HICA but not for acquiring resistance in 0% MM.
We added Supplemental Fig. S24 and an explanation (line 235).

[*(7) Are S. cerevisiae killed by auxotoxins?*
I suggest the authors examine whether S. cerevisiae are killed by auxotoxins, for example, by performing a vital dye staining.]

We performed suggested experiments by staining dead *S. cerevisiae* cells using phloxine B and confirmed that these cells were indeed killed. We added Supplemental Fig. S26 and explained this point (line 245).

[(8) *A typo*
Page 14
"cDNA libraries were prepared using the NEBNext Ultra II DNA Library Prep Kit 351 for Illumina (New England Biolabs, Inc. Ipswich, MA, USA), following the 352 manufacturer's instructions. cDNA libraries were sequenced using the Illumina 353 paired-end technology on MiSeq with MiSeq Reagent Kit v3"
In the above two sentences, I think "cDNA libraries" should be "genomic DNA libraries".]

We have corrected the typo (line 512, 514).

[(9) *How are HICA and 2K3MVA synthesized in the cells?*

The authors should discuss how HICA and 2K3MVA may be synthesized in yeast cells.]

Although synthetic pathways producing HICA and 2K3MVA have not been identified in *S. pombe*, a hydroxyacid dehydrogenase was identified as a synthetase for HICA in bacteria (Chambellon *et al.*, *J Bacteriol*, 2009 for *Lactococcus lactis*). Neither its homolog nor any enzymes for HICA and 2K3MVA synthesis have been reported in *S. pombe*. Thus, we searched genes encoding hydroxyacid dehydrogenase, whose targets are unknown in *S. pombe*, by using UniProt, and made their disruptant mutants. Then, the concentrations of HICA and 2K3MVA in media conditioned by all of these strains decreased but did not reach zero. It suggests that *S. pombe* synthesized HICA and 2K3MVA by using multiple pathways and synthetases. Further, we confirmed that the delay phase of WT cells cultured in these CM was shortened. We have added these results in the revised manuscript (Supplemental Fig. S12 and line 155).

Below we address the corrections suggested by the reviewer in a point-by-point manner. Note that the reviewer's comments are noted with brackets, and the corrections are highlighted by red in the revised manuscript.

Reviewer #4:

[*The manuscript describes results of extensive studies examining the phenomenon of voluntary triage, an yeast response to glucose starvation. This response involves the production of autotoxins which serve double functions: while a subset of cells is killed by these autotoxins, the remaining cells adapt to their presence epigenetically and can grow efficiently under conditions of glucose starvation.*

The authors find convincing evidence for this phenomenon in the related yeast S. cerevisiae and argue for a conserved mechanism. This is a well done and solid study for which I only have a few suggestions to improve the quality of the manuscript.]

Thank you for your positive evaluation of our study and constructive comments.

[1. *One major aspect of the study that I see lacking is the understanding of why some cells become resistant to HICA and 2K3MVA while others do not. And whether the fate of these subpopulations is related in any way to their ability to produce these chemicals. So would cells that produce more HICA/2K3MVA are also more resistant to them. This could be tested by testing the MIC (minimum inhibitory concentration) of mutants defective in the production of HICA/2K3MVA.*]

Although synthetic pathways producing HICA and 2K3MVA have not been identified in *S. pombe*, a hydroxyacid dehydrogenase was identified as a synthetase for HICA in bacteria (Chambellon *et al.*, *J Bacteriol*, 2009 for *Lactococcus lactis*). Neither its homolog nor any enzymes for HICA and 2K3MVA synthesis have been reported in *S. pombe*. Thus, we searched genes encoding hydroxyacid dehydrogenase, whose targets are unknown in *S. pombe*, by using UniProt, and made their disruptant mutants. Then, the concentrations of HICA and 2K3MVA in media conditioned by all of these strains decreased but did not reach zero. It suggests that *S. pombe* synthesized HICA and 2K3MVA by using multiple pathways and synthetases. Further, we confirmed that the delay phase of WT cells cultured in these CM was shortened. We have added these results in the revised manuscript (Supplemental Figs. S12 and S19, and description on lines 155 and 207).

[2.   *How specific is this effect to glucose? It would be interesting to see if the absence of other fermentative sugars would lead to a similar effect (e.g. galactose, fructose, mannose etc).*]

Thank you for your interesting suggestions.
We added various species of sugar to the CM and measured the growth of cells. We found that the behavior was different by what we added: Fructose rescued the delay as much as glucose addition, and mannose and galactose rescued partially. Those results suggest that many sugars are effective in preventing growth inhibition by autotoxins, but their effects vary with each metabolic pathway.
We added Supplemental Fig. S4 and an explanation (line 54).

[3.   *Is the effect due to glucose signaling or glucose metabolism? This can be easily tested using 2-DOG.*]

Following your suggestion, we added 2-DG into the WT CM and found that cells did not grow. It suggests that the survival of cells is due to glucose metabolism (and, moreover, sugar metabolism, considering the results above) but not glucose signaling.
We have added a result (Supplemental Fig. S4) and explained this point (line 58).

[4.   *I found the discussion on multicellularity highly speculative and somewhat unrelated to the topic. Instead, I suggest the authors allocate more space to discussing population heterogeneity and the possible reasons for why some cells are killed by or adapt to the autotoxins and the evolutionary implications of this type of selection.*]

We agreed with your suggestion. We hypothesized that the cell cycle is one of the origins generating cellular heterogeneity and confirmed it. We measured the death rate of the strain with a temperature-sensitive *cdc25-22* mutation, which can arrest the cell cycle in the G2 phase at a higher temperature. Using *cdc25-22* mutants, we prepared synchronously dividing cells and found that they showed higher death rates than asynchronously dividing cells, which will contain a substantial portion of cells arrested in the G0 phase. This suggests that the cell cycle stage is one of the important factors in determining the cell competency to survive or die, and the resting cells may show tolerance to autotoxins. We have added Supplemental Fig. S18 and a remark explaining this point to the text (line 199).
We further added the data how cells dead against the administration of the autotoxins and found that a large fraction of cells died expressing a marker for programmed cell death (Supplemental Fig. S17, line 192). The

cell cycle-related mortality and programmed cell death caused by autotoxins imply the relationship between our findings and the evolution of multicellularity. We added the description of this point (line 307).
