# Supplementary material for: Autotoxin-mediated latecomer killing in yeast communities
Source: PLoS Biol. 2022 Nov 7;20(11):e3001844. doi: 10.1371/journal.pbio.3001844 (PMC9639812; doi:10.1371/journal.pbio.3001844)
Supplement: Attachment — Submitted filename: response.pdf [file pbio.3001844.s036.pdf]

Below we address the corrections suggested by the reviewer in a point-by-point manner. Note that the reviewers' comments are noted with brackets, and the corrections are highlighted by red in the revised manuscript.

Reviewer #1:

[*This version of a manuscript is a substantial improved and more readable version, primarily establishing a biochemical basis for killing cells within a clonal population of yeast. The use of 'latecomer-killing' also seems more appropriate than involuntary triage.*

I enjoyed reading this version of the manuscript, and it is very effective as a short report. The manuscript needs a careful proofreading (to remove some minor grammatical errors, as well as slightly difficult to read sentences), but I will not suggest any further experiments.]

Thank you for your positive evaluation of our manuscript.
We have corrected some grammatical errors following your suggestion.

[*The last paragraph of the discussion (on the origins of multicellularity) is overly suggestive (especially with the liberal use of 'imply'). Of course, some speculation is encouraged in the discussion, but for example "The cell cycle dependency of cell death (Fig. S18) and the apoptotic pathway activation in a large fraction of dead cells (Fig. S17) also imply a relationship between latecomer killing and multicellularity." is quite optimistic, since it is still not clear how meaningful these data are with respect to say the artificial experiments forming snowflake yeasts. I would recommend a careful toning down of the last paragraph, but without eliminating the speculative element.*]

We have toned down the last paragraph following your suggestion.

Reviewer #2:

[*In this revision, the authors have converted their paper to a short report focused on the phenomenon of toxin release (identified here using mass spectrometry as primarily leucic acid--2-hydrozyisocaproic acid, HICA and L-2keto-3methyvalerate--2K3MVA) under glucose starvation conditions which they now term "latecomer killing". In addition, they have performed additional important and interesting experiments including measuring HICA and 2K3MVA concentration in conditioned media, assessing additional mutants,*

assessing additional nutrient add-backs, performing a 2-DOG experiment, analyzing cell death, and determining the effect of the cell cycle on killing.]

Thank you for your positive evaluation of our manuscript.

[*The manuscript is improved over the previous version and functions better as a short report. Indeed, the observation of the autotoxin phenomenon is extremely interesting and worth reporting. However, although the authors have performed additional experiments questions remain about whether the phenomenon provides a population-level advantage, why there is so much variability in the mechanism of cell death (if being caused by the same autotoxins), and mechanistically what is causing resistance or lack there-of. In addition, while the authors have dialed back some of their language the connections to cheater dynamics and multicellularity are tenuous at best. In addition, there is a tendency to oversell the results (for example, while the cell cycle data is intriguing it does not directly show that it is G0 cells that are resistant and indeed the idea that it is G0 cells also somewhat contradicts that idea that there is a dividing, resistant population.) Furthermore, lines 63-69*
suggest that cells are inheriting an adapted state because their growth rate remains constant in CM. Since adapted cells are being inoculated into CM, I am unconvinced of this explanation. Of course, there is continuous pressure for cells to maintain the adapted state. That doesn't mean that they are necessarily remembering it as they divide, particularly in the epigenetic sense (although the authors did remove reference to epigenetics).

This paper reports a very interesting phenomena that is deserving of publication with interesting experiments that hint at potential mechanisms and population-level effects. However, in some places the results are oversold and mechanistic insight is lacking.]

Following your suggestion, we have toned down several points in the manuscript.

Reviewer #3:

[*The revision has addressed all my concerns. I support the publication of this paper.*]

Thank you for your positive evaluation of our manuscript.

Reviewer #4:

[*The authors have addressed most of the points raised by all 4 reviewers, either through additional experiments or through changes to the text.*

Some issues could not be addressed due to the extensive work required which would be greatly beyond the scope of a Short Report.

The results with the different sugars and the cell cycle mutant are interesting and worth pursuing.]

Thank you for your positive evaluation of our manuscript.
